# Designed miniproteins potently inhibit and protect against MERS-CoV

**DOI:** 10.1016/j.celrep.2025.115760

**Published:** 2025-05-31

**Authors:** Robert J. Ragotte, M. Alejandra Tortorici, Nicholas J. Catanzaro, Amin Addetia, Brian Coventry, Heather M. Froggatt, Jimin Lee, Cameron Stewart, Jack T. Brown, Inna Goreshnik, Jeremiah N. Sims, Lukas F. Milles, Basile I.M. Wicky, Matthias Glögl, Stacey Gerben, Alex Kang, Asim K. Bera, William Sharkey, Alexandra Schäfer, Jack R. Harkema, Ralph S. Baric, David Baker, David Veesler

**Affiliations:** 1 Department of Biochemistry, University of Washington, Seattle, WA 98195, USA.; 2 Institute for Protein Design, University of Washington, Seattle, WA 98195, USA.; 3 Department of Epidemiology, University of North Carolina, Chapel Hill, NC 27599, USA.; 4 Department of Pathobiology and Diagnostic Investigation, Michigan State University, East Lansing, MI 48824, USA.; 5 Howard Hughes Medical Institute, University of Washington, Seattle, WA 98195, USA.

**Keywords:** protein design, cryo-EM, X-ray crystallography, MERS-CoV, MERS-CoV S, infectious disease, structural biology

## Abstract

Middle East respiratory syndrome coronavirus (MERS-CoV) is a zoonotic pathogen with 36% case-fatality rate in humans. No vaccines or specific therapeutics are currently approved to use in humans or the camel host reservoir. Here, we computationally designed monomeric and homo-oligomeric miniproteins binding with high affinity to the MERS-CoV spike (S) glycoprotein, the main target of neutralizing antibodies and vaccine development. We show that these miniproteins broadly neutralize a panel of MERS-CoV S variants, spanning the known antigenic diversity of this pathogen, by targeting a conserved site in the receptor-binding domain (RBD). The miniproteins directly compete with binding of the DPP4 receptor to MERS-CoV S, thereby blocking viral attachment to the host entry receptor and subsequent membrane fusion. Intranasal administration of a lead miniprotein provides prophylactic protection against stringent MERS-CoV challenge in mice motivating future clinical development as a next-generation countermeasure against this virus with pandemic potential.

## In brief

Ragotte, Tortorici, Catanzaro *et al.* de novo designed potent inhibitors of MERS-CoV entry into cells. These homotrimeric molecules engage multiple copies of the MERS-CoV RBD on the spike protein to block engagement with host receptor DPP4, neutralize diverse MERS-CoV strains and prophylactically protect against MERS-CoV disease *in vivo* when administered intranasally.

## Graphical Abstract

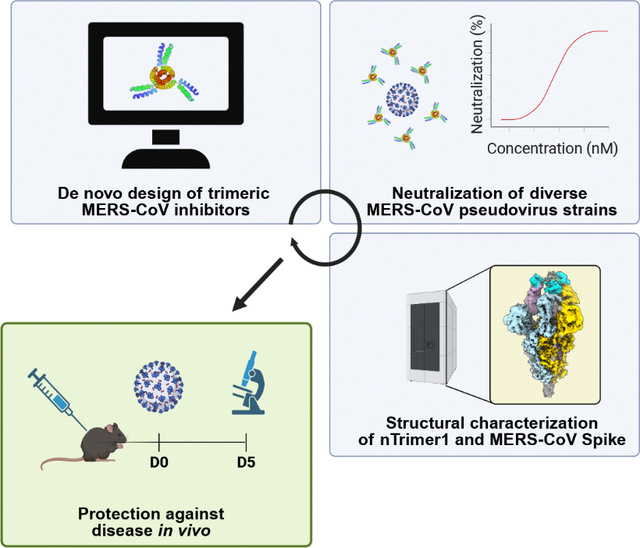

## Introduction

Middle-East respiratory syndrome coronavirus (MERS-CoV) is a betacoronavirus causing severe and often deadly respiratory disease that was first identified in Saudi Arabia in 2012^1,2^. MERS-CoV is a zoonotic virus, and most human cases result from direct viral transmission from the dromedary camel host reservoir or from human-to-human transmission in healthcare settings. Between April 2012 and August 2024, the European Centre for Disease Prevention and Control reported a total of 2,622 laboratory-confirmed MERS cases and 953 deaths globally, corresponding to a fatality rate of 36%^3^. MERS-CoV is endemic in camels in the Arabian Peninsula^4^ and has recently been detected in humans in Africa^5^, underscoring possibly wider distribution and transmission than previously appreciated. To date, no vaccines or specific therapeutics are approved to prevent or treat MERS-CoV infections in humans or animals.

Entry of MERS-CoV into susceptible cells is mediated by the spike (S) glycoprotein which forms homotrimers protruding from the viral envelope^6–8^. S is the major antigen present at the viral surface and is the target of neutralizing antibodies during infection as well as the focus of vaccine design^7,9–11^. MERS-CoV S comprises an S_1_ subunit, which mediates binding to sialosides via the N-terminal domain (NTD) and to the dipeptidyl-peptidase 4 entry receptor (DPP4) via the receptor-binding domain (RBD), as well as an S_2_ subunit which promotes virus and host membrane fusion to initiate infection^,12–17^. The differential distribution of these receptors in humans (lower airways) and camels (upper airways) possibly explains the severity but limited transmissibility of MERS-CoV in humans and the milder but more infectious profile in the reservoir host^14,18^. The continued circulation of MERS-CoV and related merbecoviruses^19–23^ and the risk of emergence of more transmissible strains motivate the development of potent and scalable therapeutics for pandemic preparedness. Computationally designed miniproteins are emerging as competitive alternatives to monoclonal antibodies due to their high binding affinity, cost-effective manufacturing and the possibility to administer them directly in the upper respiratory tract (i.e. the initial site of infection for respiratory pathogens). Here, we set out to computationally design miniproteins targeting the MERS–CoV RBD and characterized the molecular basis of binding and inhibition, neutralization potency and breadth and *in vivo* prophylactic protection.

## Results

### Design of MERS-CoV RBD-directed miniproteins

**Fig 1.**
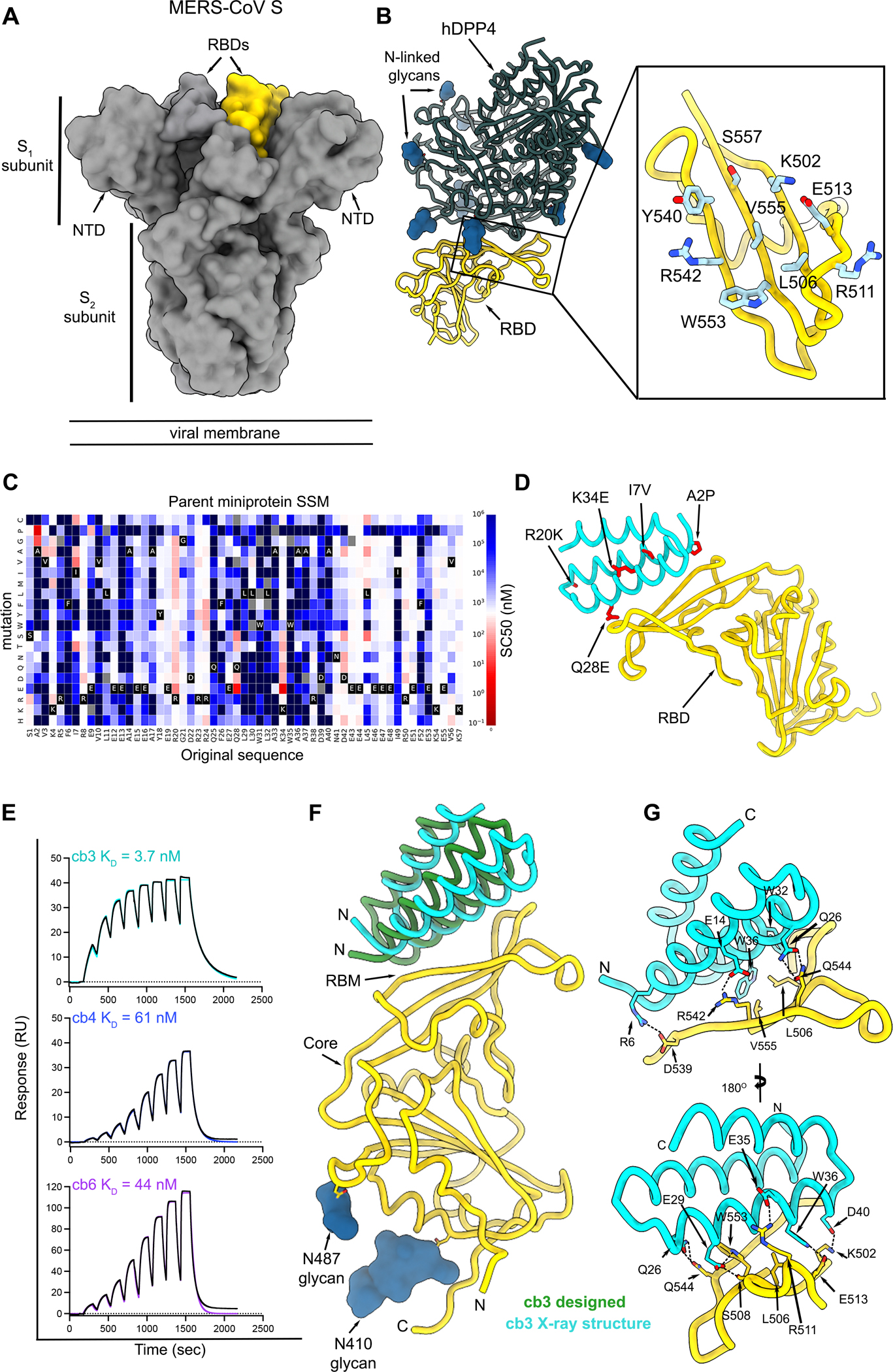
Computational design of MERS-CoV S RBD-targeting miniproteins. **A.** Surface representation of the prefusion MERS-CoV S trimer (grey, PDB 6Q04) highlighting the position of a single RBD in yellow. N-linked glycans are omitted for clarity. **B.** Ribbon representation of human DDP4 (dark green) in complex with the MERS-CoV S RBD (yellow) (PDB 4L72). Inset: zoomed-in view of the MERS-CoV S receptor-binding motif (RBM) highlighting the residues targeted for miniprotein design. **C.** Site saturation mutagenesis of the initial hit, designated parent miniprotein. For each point mutation, the concentration that achieves 50 % of the saturating binding signal on yeast (SC50^32^) was calculated and plotted as enhancing affinity (red), reducing affinity (blue) or not affecting affinity (white) as compared to the SC50 of the parental design. The amino acid residue identity at each position of the parent miniprotein is colored black with white text, with all possible amino acid substitutions for that position following the y-axis. Gray indicates mutations that were not present in the library. **D.** Selected mutations from the parent miniprotein to yield the cb3 design are indicated on the ribbon diagram of the complex X-ray structure. **E.** Evaluation of binding of the cb3, cb4 and cb6 designed miniproteins to biotinylated MERS-CoV S EMC/2012 RBD immobilized on a biotin capture chip using surface plasmon resonance (SPR). Eight miniprotein concentrations were used starting at 500 nM and following a two-fold dilution series using single cycle kinetics. RU: response units. Binding data and fits are shown as black and colored lines, respectively **F.** Superimposition of the cb3-bound MERS-CoV S RBD (gold) from the computationally designed model (green) and the experimental crystal structure (cyan). Only one RBD is shown for clarity. **G.** Zoomed-in views of the crystal structure of the complex between cb3 (cyan) and the MERS-CoV S RBD (gold) highlighting selected interactions. Dashed lines indicate hydrogen bonds and salt bridges.

Given that RBD-directed antibodies account for most plasma neutralizing activity in humans previously infected with SARS-CoV-2 or MERS-CoV^9,28–30^, we targeted this domain to design MERS-CoV miniprotein inhibitors. We used Rosetta-based methods to target hydrophobic residues within the MERS-CoV S receptor-binding motif (RBM) - hDPP4 interface through docking of a three-helix bundle library using RifDock^25,31^ (Fig 1A–B). FastDesign was used for sequence-design of these scaffolds followed by filtering based on contact molecular surface and Rosetta ΔΔG. The best interface motifs (according to ΔΔG) were grafted onto new backbones using MotifGraft^32^ and designs that bound to the biotinylated MERS-CoV S EMC/2012 RBD *via* yeast-surface display were subsequently optimized using site-saturation mutagenesis to identify affinity-enhancing mutations (Fig 1C–D). The process yielded three constructs, designated cb3, cb4 and cb6, with respective binding affinities for the MERS-CoV S EMC/2012 RBD of 3.7 nM, 61 nM and 44 nM, as determined by surface-plasmon resonance (SPR) (Fig 1E).

**Fig 2.**
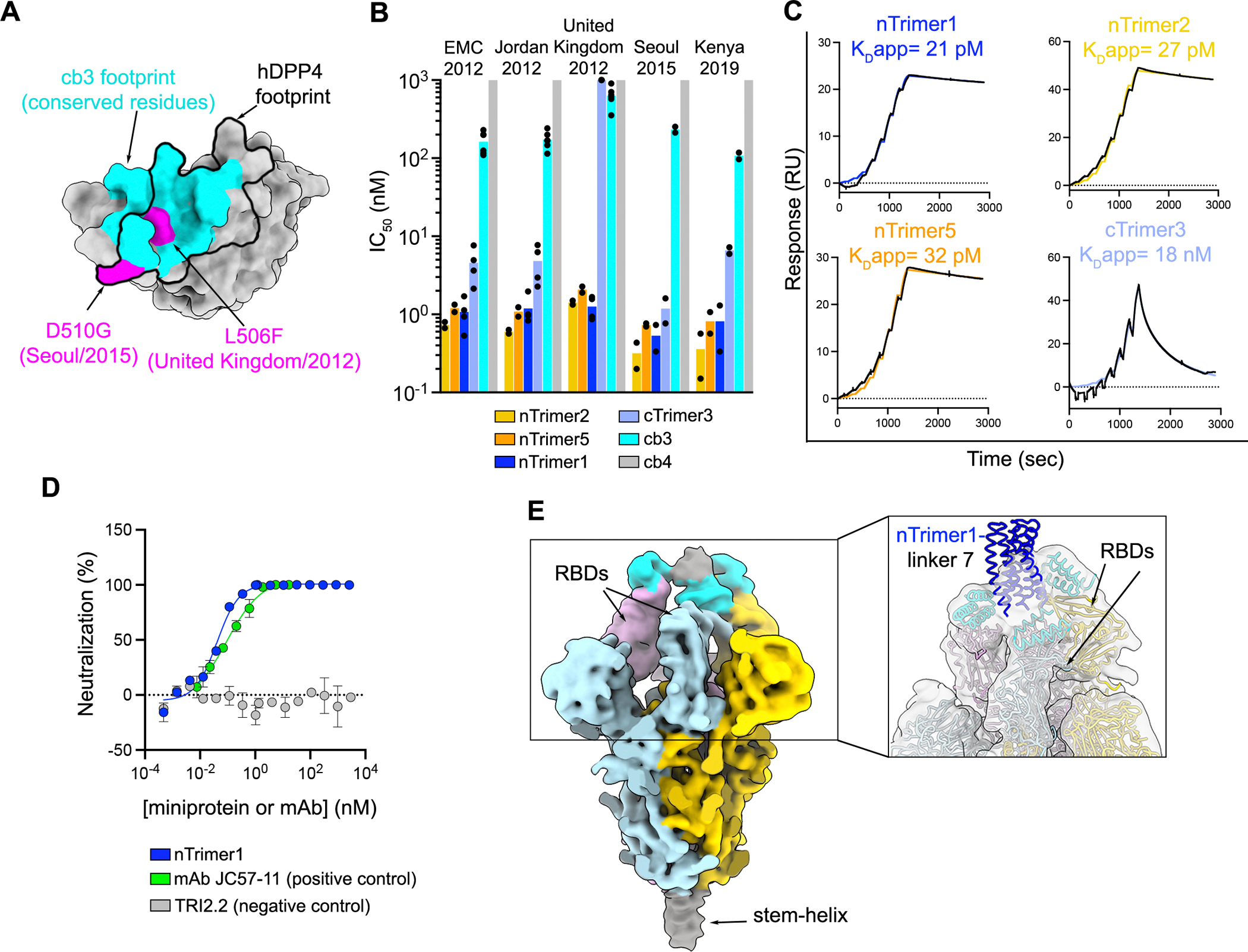
Miniprotein homotrimerization enhances neutralization potency. **A.** Surface representation of the MERS-CoV EMC/2012 RBD highlighting in cyan the conserved residues in the cb3-binding site among MERS-CoV EMC/2012, Jordan/2012, United Kingdom/2012, Seoul/2015 and Kenya/2019. The L506F and D510G residue substitutions present in the United Kingdom/2012 and Seoul/2015 isolates, respectively, are shown in pink whereas the hDPP4 footprint is shown as a black outline. **B.** Neutralizing activity of monomeric and trimeric miniproteins against a panel of MERS-CoV S variant VSV pseudoviruses. cb4, for which we did not detect neutralization, was plotted at the limit of detection (LOD) of 10^3^ nM. Each dot represents an IC_50_ expressed in nM obtained from a biological experiment. Bars represent the mean of the IC_50_s. Each biological experiment was performed with technical duplicates and curves are shown in Fig. S1 and S2. The IC_50_s plotted here are listed in Table 1 and Table S2. **C**. SPR analysis of binding of trimeric cb3 constructs to the prefusion biotinylated MERS-CoV EMC/2012 S ectodomain trimer immobilized on biotin capture chips. Single cycle kinetic analysis of a two-fold dilution series starting at 8 nM of trimer is shown except for cTrimer3 which had an upper concentration of 167 nM. Binding data and fits are shown as black and colored lines, respectively. **D**. Neutralization of MERS-CoV EMC/2012 authentic virus in the presence of various dilutions of the indicated miniproteins and JC57–11 mAb. TRI2.2 miniprotein and JC57–11 mAb were used as negative and positive controls, respectively. A single biological experiment is shown where the error bars represent the standard error of the mean (SEM) of technical duplicates. **E.** CryoEM reconstruction of the nTrimer1_linker 7 bound to prefusion MERS-CoV S which is low-pass filtered at 8 Å resolution and colored by protomer. The density corresponding to three bound cb3s is colored in cyan and the remaining density corresponding to the trimerization domain is colored gray. Inset: zoomed-in view of a model depicting nTrimer1_linker 7 (cb3-GGGSGGGS-SB175) fitted into the map shown in panel D. cb3: cyan; trimer1: navy. The linkers were not modeled.

To characterize the molecular basis of MERS-CoV S recognition by these miniproteins, we determined a crystal structure of the RBD in complex with cb3 at 1.85 Å resolution using X-ray diffraction data (Table S1). cb3 interacts with the RBM through polar interactions and shape complementarity burying an average surface of 750 Å^2^ at the interface between the two binding partners (Fig 1F–G and Fig 2A). cb3 utilizes its N-terminal two helices to interact with the RBD residues 502, 506–508, 511–513, 536–542, 544, 552–553, 555 without contacts with N-linked glycans. cb3 residues R6, E14, E35 and D40 are salt bridged to RBD residues D539, R542, R511 and K502, respectively, and a constellation of hydrogen bonds and van der Waals interactions strengthen the binding interface (Fig 1F–G). Superimposition of the RBD from the crystal structure determined here with that of the computationally designed complex reveals that the cb3 miniprotein aligns with an RMSD of 4.8 Å for 57 aligned Cα positions (Fig 1F).

### Monovalent and multivalent miniproteins broadly neutralize MERS-CoV variants

To evaluate the neutralization potency and breadth of the monomeric miniproteins, we quantified their dose-dependent inhibitory activity using vesicular stomatitis virus (VSV) particles pseudotyped with a panel of MERS-CoV S variants (see methods). All of these variants were identified in humans except for MERS-CoV Kenya/2019 which was detected in a camel^33^. Relative to EMC/2012 S, these variants comprise 2–8 amino acid mutations with United Kingdom/2012 S and Seoul/2015 S harboring residue changes known to reduce the neutralizing activity of monoclonal antibodies and polyclonal sera^34–36^. Out of the three monovalent miniproteins designed, only cb3 had detectable neutralizing activity against MERS-CoV EMC/2012 S VSV, likely due to differences in overall binding affinity which is an order of magnitude greater for cb3 relative to cb4 and cb6 (Fig 1E and Fig 2B). The neutralization IC_50_s and binding kinetics for all three monomers are included in Table S2 and Table S3, respectively. cb3 broadly inhibited MERS-CoV pseudoviruses harboring all S variants from our panel with up to 4.5-fold reduced potency for the United Kingdom/2012 isolate, compared to EMC/2012, most likely due to the L506F substitution within the cb3 binding site (Fig 2B, full curves for each replicate in Fig S1).

Based on our previous success in designing a homotrimeric, pan-variant SARS-CoV-2 inhibitor, designated TRI2–2^24–27^, we set out to engineer homotrimeric versions of cb3 to enable simultaneous engagement of all three RBDs within a MERS-CoV S trimer and enhance potency and breadth further through avidity. We developed multivalent miniproteins using a set of validated trimerization motifs^24,37^ fused to the cb3 N or C terminus *via* a GSG linker. 12 trimeric cb3 constructs were designed, consisting of 6 oligomerization domains (Table S4) with both N- and C-terminal fusions for each oligomerization domain (denoted with prefix ‘n’ or ‘c’ to describe the position of the miniprotein relative to the trimerization domain). Two of these failed to express (nTrimer3 and nTrimer4). Evaluation of neutralizing activity against the MERS-CoV EMC/2012, Jordan/2012 and United Kingdom/2012 pseudoviruses revealed that trimeric cb3 miniproteins were endowed with at least an order of magnitude greater potency, compared to monomeric cb3, with the exception of cTrimer1, cTrimer3, cTrimer4, cTrimer5 and cTrimer6 fusions which failed to neutralize MERS-CoV United Kingdom/2012 S VSV pseudotypes (Table S2,full curves for each replicate in Fig S1). SPR analysis of binding to the immobilized prefusion MERS-CoV S for the most potently neutralizing homotrimers (nTrimer1, nTrimer2 and nTrimer5) alongside one inferior homotrimer (cTrimer3) showed that multimerized constructs with an N-terminal miniprotein and C-terminal oligomerization domain interacted with higher apparent affinities and had markedly reduced off-rates relative to monomeric cb3, consistent with avid binding, thus explaining the improved neutralization (Fig 2C with measured kinetics parameters in Table S3). We therefore selected the nTrimer1, nTrimer2 and nTrimer5 homotrimeric miniproteins for further characterization of neutralization breadth and observed that all three of them inhibited the MERS-CoV Seoul/2015 and Kenya/2019 S VSV pseudoviruses, markedly outperforming monomeric cb3-mediated neutralization (Fig 2B, full curves for each replicate in Fig S2 and associated IC_50_s in Table 1). We next showed that Trimer1 neutralized authentic MERS-CoV EMC/2012 with an IC_50_ of 40 pM, comparable to the control mAb JC57–11^38^ (Fig 2D). Storage and shipping conditions are key factors affecting the development of therapeutic molecules as they can impact the cost of manufacturing,distribution and access. Lyophilization has emerged as a method of storage and administration of protein biologics to increase shelf life and optimize *in vivo* release^39,40^. We found that *in vitro* neutralization potency and breadth against our panel of MERS-CoV S variants were unaffected by freeze/thawing or lyophilization/reconstitution of nTrimer1, underscoring the optimal biochemical properties of this miniprotein (Fig S2C).

CryoEM characterization of nTrimer1 bound to prefusion MERS-CoV EMC/2012 S revealed that the observed enhancement of avidity and neutralizing activity afforded by homotrimerization results from engagement of two or three RBDs within each trimer (Fig S3, collection and refinement statistics in Table S5). We subsequently produced nTrimer1 homotrimeric constructs harboring systematic variations of linker length and evaluated their neutralizing activity against our panel of MERS-CoV S variants (Fig S4A with linker sequences in Table S4). Given that the two constructs harboring the longest linkers, designated nTrimer1_linker 7 (cb3_GGGSGGGS_SB75) and nTrimer1_linker 8 (cb3_GGGSGGGSGGGS_SB175), exhibited slight improvements in neutralization potency (Fig S4A), we determined an asymmetric cryo-EM reconstruction of the former miniprotein construct bound to MERS-CoV S at 2.6 Å resolution, which also indicated variable minibinder stoichiometry among S trimers (Fig 2E, with data processing pipeline in Fig S5 and refinement statistics in Table S5). Subsequent local refinement of the region corresponding to two neighboring MERS-CoV S RBDs each bound to a cb3 module yielded a reconstruction at 3.2 Å resolution that was virtually indistinguishable from the crystal structure of the monomeric cb3-bound MERS-CoV S RBD (Fig S4B). These results indicate that oligomerization enabled retention of the binding mode designed.

Collectively, these data show that the multimeric miniproteins potently inhibit MERS-CoV variants known to evade monoclonal antibody-mediated neutralization and are hyperstable,two highly desirable properties for the development of countermeasures against coronaviruses.

### The nTrimer1 miniprotein interferes with MERS-CoV S binding to the host receptor

**Fig 3.**
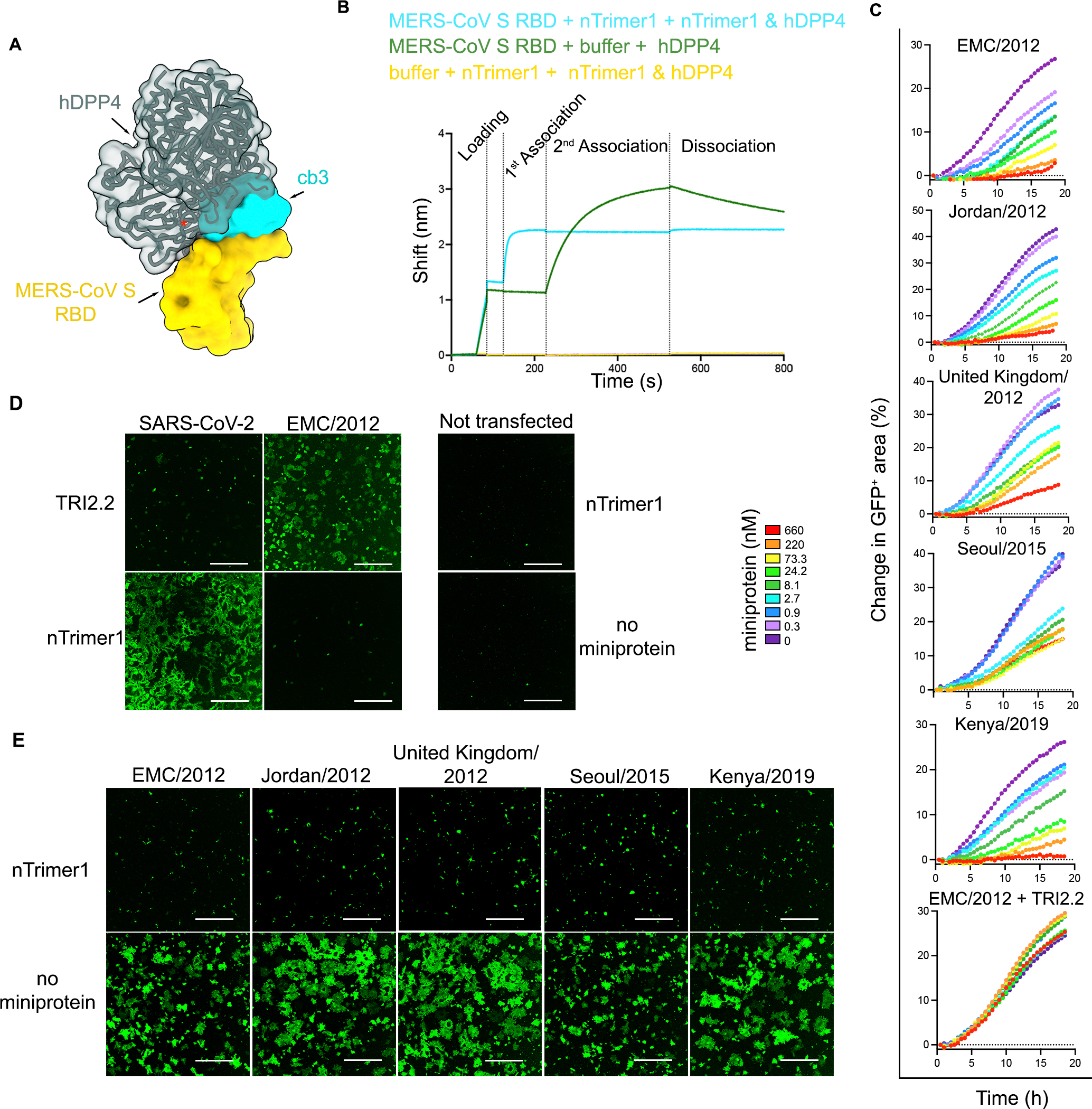
nTrimer1 inhibits MERS-CoV attachment to the hDPP4 receptor. **A**. Composite model showing that the miniprotein cb3 (cyan surface) and human DPP4 (hDPP4, dark green) bind to partially overlapping binding sites on the MERS-CoV RBD (rendered as a yellow surface). The red asterisk indicates steric clashes. **B.** BLI analysis of hDPP4 binding to MERS-CoV S RBD. Streptavidin biosensor associated with biotinylated MERS-CoV S RBD were dipped in a solution containing either 0.5 μM of nTrimer1 or buffer (1^st^ association) and subsequently in a solution containing 0.5 μM of hDPP4 and 0.5 μM of nTrimer1 (cyan) or only 0.5 μM of hDPP4 (green) (2^nd^ association). Streptavidin biosensors with no RBD associated were used as a negative control (yellow). **C-E**. Kinetics of cell-cell fusion (expressed as a change in percent of GFP^+^ area) promoted by the different MERS-CoV S glycoproteins over an 18 h time course experiment using a split GFP system in the presence or absence of different concentrations of nTrimer1. BHK-21 effector cells transfected with SARS-CoV-2 (Wuhan-Hu-1) S or MERS-CoV (EMC/2012) S both incubated with nTrimer1 and TRI2.2 were used as a specificity control. Not transfected cells were used as negative controls **(D)**. nTrimer1-mediated inhibition of cell-cell fusion (at 660 nM) between effector BHK-21 cells transiently transfected with MERS-CoV S variants and target VeroE6-TMPRSS2 **(E).** Images correspond to the end point of the kinetic experiment. Scale bars: 1000 μm. Data represent one experiment out of two biological replicates.

Our structural data suggest that cb3 binding to the RBD would be incompatible with engagement of the hDPP4 receptor (Fig 3A). We assessed whether nTrimer1 and hDPP4 can simultaneously bind to the RBD using biolayer interferometry (BLI). We confirmed that nTrimer1 binding to the immobilized RBD blocked subsequent binding of hDPP4 (Fig 3B). We next investigated whether nTrimer1 impacted cell-cell fusion mediated by the MERS-CoV EMC/2012, United Kingdom/2012, Jordan/2012, Seoul/2015, and Kenya/2019 S variants (Fig 3 C–E). We recorded live cell imaging using a split green fluorescent protein (GFP) system using VeroE6 target cells stably expressing human TMPRSS2 and GFP_11_ and BHK-21 effector cells stably expressing GFP_1–10_ and transiently transfected with a MERS-CoV S variants^41^. We monitored cell-cell fusion over a period of 18 h in the presence of different concentrations of nTrimer1 and observed dose-dependent inhibition of membrane fusion which was completely abrogated at a concentration of 660 nM (Fig 3C–E). The SARS-CoV-2 S-directed TRI2–2 miniprotein, which uses the same trimerization domain^24^ as nTrimer1, had no effect on MERS-CoV S-mediated fusion, confirming the specificity of the inhibition observed (Fig 3C, 3D). These data indicate that the neutralizing activity of nTrimer1 against MERS-CoV S variants results from direct competition with viral attachment to the host cell receptor DDP4, which in turn blocks membrane fusion.

### nTrimer1 protects mice against MERS-CoV challenge

**Fig 4.**
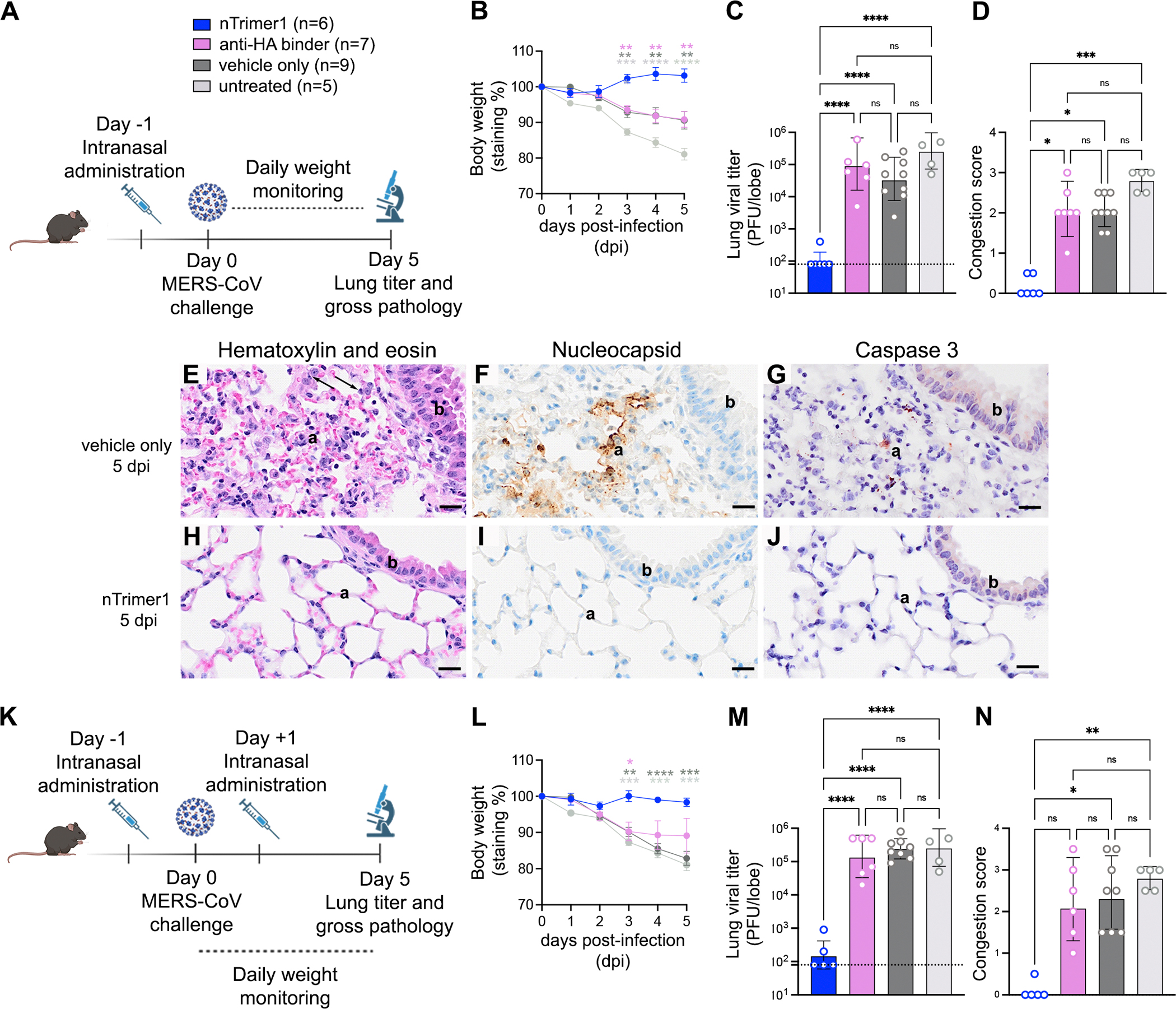
Intranasal administration of nTrimer1 protects mice against MERS-CoV challenge. **A.** Study design. nTrimer1, monomeric influenza hemagglutinin miniprotein (anti-HA) or buffer alone (TBS) were administered intranasally to C57BL/6 J288/330 mice (6.25 mg/kg) one day prior to challenge with MERS-CoV-m34c5. An untreated group was also included. **B**. Weight loss at different days post infection (dpi). The error bars represent the standard error of the mean (SEM) of technical duplicates. **C**. Lung viral titers at 5 dpi. Bars represent the geometric mean and error bars the standard error of the geometric mean. Limit of detection is indicated with a dashed line. **D**. Congestion score at 5 dpi. Bars represent the geometric mean and error bars the standard error of the geometric mean **(E–J).** Pulmonary histopathology analysis of light photomicrographs of lung sections at 5 dpi stained with hematoxylin and eosin **(E and H)**, polyclonal anti-MERS-CoV nucleocapsid mouse sera (marker for intracellular viral replication) and counterstained with hematoxylin **(F and I)**, or Caspase 3 (marker for apoptosis) and counterstained with hematoxylin **(G and J).** The analysis was carried out for mice administered with vehicle only (TBS) **(E-G)** or nTrimer1 **(H-J)**. Alveolar septa, a; bronchiolar epithelium, b; alveolar type 2 cell hyperplasia, solid arrows. Scale bar: 20 μm. **K.** Study design. nTrimer1, influenza hemagglutinin miniproteins (anti-HA), or vehicle only were administered to mice C57BL/6 J 288/330 at −1 and +1 dpi. An untreated group was also included. **L.** Weight loss at different dpi. The error bars represent the standard error of the mean (SEM) of technical duplicates **M**. Lung viral titers at 5 dpi. Bars represent the geometric mean and error bars the standard error of the geometric mean. Limit of detection is indicated with a dashed line. **N**. Congestion score at 5 dpi. Bars represent the geometric mean and error bars the standard error of the geometric mean. Group comparisons for body weight and viral titers were assessed with the two-way ANOVA: Tukey’s test. For the congestion score, comparisons among groups were assessed with the Kruskal-Wallis test; ns, not significant; *P < 0.05, **P < 0.01, ****P < 0.0001.

To evaluate the *in vivo* protective efficacy of one of these homotrimeric miniproteins, we inoculated susceptible C57BL/6J 288/230 mice^42^ with 10^5^ p.f.u. MERS-CoV-m34c5 intranasally and followed weight loss for 5 days as a proxy for disease. nTrimer1 was administered intranasally at 6.25 mg/kg one day before challenge and control groups were treated identically except that they received an influenza virus hemagglutinin-directed miniprotein, buffer alone (TBS), or were untreated (Figure 4A). Prophylactic administration of nTrimer1 completely protected challenged mice against weight loss throughout the duration of the experiments and abrogated viral replication in the lungs. In contrast, control groups experienced 10–20% weight loss and had ~3 orders of magnitude greater lung viral titers, relative to animals receiving nTrimer1 (Figure 4B–C). Gross pathological assessment of lung tissues at 5 days post viral challenge revealed that nTrimer1 prophylaxis entirely protected from visible signs of lung hemorrhage, as opposed to control groups that uniformly exhibited lung discoloration induced by MERS-CoV (Figure 4D). Immunohistochemical staining for MERS-CoV nucleocapsid (N+) and caspase 3 (cas3+) assessed viral replication and apoptosis, respectively, alongside hematoxylin and eosin stained sections for general histopathology. At 5 dpi, vehicle control (TBS + virus) mice had subacute proliferative alveolitis with alveolar type 2 cell hyperplasia, mononuclear cell inflammation (monocytes and lymphocytes), and scattered N+ and cas3+ cells (Figure 4E–G). In contrast, nTrimer1-treated mice had no N+ or cas3+ cells and no other pulmonary histopathology (Figure 4H–J). Mice administered with nTrimer1 before and after MERS-CoV challenge, were also protected from weight loss, lacked gross pathological lung abnormalities and had significantly reduced viral titres in the lung, mirroring, but not improving upon, the results of prophylactic administration alone (Figure 4K–N). These data show that a single dose of nTrimer1 administered one day prior to viral exposure provides prophylactic protection against a stringent MERS-CoV challenge and reduces lung viral burden and histopathological lesions.

## Discussion

A comprehensive pandemic preparedness strategy should include the development of high-throughput medical intervention platforms that can rapidly curtail transmission and lethality of priority pathogens during outbreaks, as opposed to relying on new medicines developed only once the pandemic has started. Emergency drug-use authorizations took months to years to be granted during the COVID-19 pandemic for prophylactic and therapeutic monoclonal antibodies developed against SARS-CoV-2^11,43^. An ideal MERS-CoV antiviral would inhibit a wide diversity of MERS-CoV variants, protect against disease, limit transmission, enable cost-effective and scalable manufacturing and distribution, while having a minimally invasive route of administration. Intranasally administered countermeasures are especially attractive as they are delivered directly at the site of initial viral infection and they can act immediately, without delays to mount an immune response, in contrast to vaccines.

The nTrimer1 miniprotein MERS-CoV inhibitor developed in this work is an excellent candidate for such pre-pandemic MERS antiviral development. nTrimer1 blocks interactions of MERS-CoV S with the DPP4 entry receptor and provides prophylactic protection against disease by reducing viral burden in the lungs by greater than three orders of magnitude. The broadly neutralizing activity of nTrimer1 against a panel of MERS-CoV S variants demonstrates some degree of resilience to viral evolution and antigenic changes, as observed for SARS-CoV-2 variants throughout the COVID-19 pandemic^41,44–48^. De novo SARS-CoV-2 miniprotein monomers were susceptible to viral escape across different SARS-CoV-2 isolates^24^. These isolates are comparatively more diverse than the MERS-CoV isolates tested here. While we show cross-neutralization between different MERS-CoV isolates, in the event of increased transmission and diversification of the virus, it is possible these minibinders would be similarly susceptible to escape. This is mitigated in part by trimerization, as it has been shown that this can overcome substantial loss in monomer affinity to retain neutralizing activity in the case of SARS-CoV-2^27^.

Given the threat that MERS-CoV poses to global biosecurity, the continued clinical development of this molecule should assess its ability to accelerate recovery when administered solely after exposure as well as its ability to limit transmission. The development of viral inhibitors, assessment of their safety, manufacturing and stockpiling prior to the emergence of a pandemic variant of a given virus is a compelling approach to ensure medical countermeasures are available for emerging and re-emerging threats.

### Limitations of this study

While we show protection *in vivo* when nTrimer1 is administered prophylactically, we did not test nTrimer1 efficacy if administered after infection with MERS-CoV. Given the importance of therapeutic efficacy post-exposure, we will evaluate nTrimer1 efficacy in this context. Furthermore, while we show effective pseudovirus neutralization against MERS-CoV isolates, greater breadth covering ACE2 dependent bat-borne merbecoviruses would be desirable in the event of zoonotic spillover.

## Resource availability

### Lead contact

Requests for further information, resources or reagents should be directed to the lead contact, David Veesler (dveesler@uw.edu).

### Materials availability

The amino acid sequences for all designs tested in this paper are provided in Table S4. The expression plasmids used for design screening (LM0627, petcon3) have been previously deposited on addgene (191551, 41522). All materials generated for this study are available from the lead contact, upon reasonable request.

### Data availability

- Structural data has been deposited to the PDB and EMDB with the entry codes listed in Tables S1 and S5
- This paper does not contain original code
- Additional information required for further analysis or re-analysis is available through the lead contact upon reasonable request

## Star Methods

### EXPERIMENTAL MODEL AND STUDY PARTICIPANT DETAILS

#### In vivo animal studies

Animal husbandry and all *in vivo* experiments were performed at the University of North Carolina at Chapel Hill with prior approval from the Institutional Animal Care and Use Committee (IACUC protocol: 22–184) in a Biosafety Level 3 (BSL-3) laboratory. 25- to 28-week-old male and female 288/330 mice on a C57BL/6J background^42^ were used for all experiments. All mice were uninvolved in previous procedures, with no prior infections and were confirmed to be healthy with comparable body weights across groups. Animals were group-housed by sex in cages under standard laboratory conditions, including a 12-hour light/dark cycle and ambient temperature (20–24°C). Mice were provided *ad libitum* access to a standard laboratory rodent diet and water. Littermates of the same sex were randomly assigned to experimental groups.

#### Cell lines

Cell lines used in this study were obtained from ATCC (HEK293T, female), JCRB-Cell Bank (VeroE6-TMPRSS2, female), Thermo Fisher (Expi293F^™^ cells, female), generated in the lab (BHK-21-GFP_1–10_ (male), or VeroE6-TMPRSS2-GFP_11_), or were gifted from Dr. Charles Rice (Rockefeller University, Huh7.5, male). Cells were cultivated at 37°C, in an atmosphere of 5 % CO_2_ and with 130 rpm of agitation for suspension cells. None of the cell lines used were routinely tested for mycoplasma contamination nor authenticated.

### METHOD DETAILS

#### Plasmids

All genes for mammalian expression synthesized by GenScript, codon optimized for expression in mammalian cells and in frame with a Kozak’s sequence to direct translation, except for the plasmid encoding membrane-anchored MERS-CoV EMC/2012 S (NC_019843.3), which was kindly provided by Whittaker lab. Genes encoding MERS-CoV RBD and MERS-CoV-2P Spike (S) ectodomain were cloned into pCMVR and pcDNA3.1 (+), respectively, with the signal peptide derived from the μ phosphatase: MGILPSPGMPALLSLVSLLSVLLMGCVAETGT. MERS-CoV RBD gene was fused at the c-terminus to an octahistidine tag while MERS-2P S protein was c-terminally fused to a foldon trimerization motif followed by a hexahistidine tag for affinity purification. Gene encoding human DPP4 was cloned in pDNA3.1 (+) with the signal peptide from the μ phosphatase and c-terminally fused to thrombin cleavage site followed by an FC-tag used for purification. Genes encoding MERS-CoV S full-length strains, 2cJordan-N3/2012 (residues 1 to 1353, AHY21469.1), United Kingdom/2012 (residues 1–1353, NC_038294.1), Seoul/2015 (residues 1–1353, KT374056.1 OK094446.1) and Kenya/2019 (residues 1–1353) strains were untagged and with the native signal peptide. All the mutations in the MERS_CoV S variants used in this study relative to MERS-CoV EMC/2012 S were described in Addetia *et al.,* 2024 except for 2cJordan-N3/2012, hereafter named MERS-CoV S Jordan/2012, which harbor the following substitutions relative to EMC/2012: G94V, H194Y, L301R, I879T, A1158S.

Monomeric cb3, cb4 and cb6 were ordered as eblock fragments and inserted into the LM0627 plasmid (Aggene 191551) flanked by the MSG residues at the N-terminal and by a SNAC^50^ and his-tag at the C terminus. We used a BsaI golden gate assembly (NEB) in a 1 μl mixture reaction containing: 0.1 μl water, 0.1 μl T4 ligase buffer, 0.375 μl eblock fragment at 4 ng/μl, 0.06 μl of BsaI-HFv2, 0.1 μl T4 ligase, 0.275 μl of LM0627 vector at 50 ng/μl. Reactions were incubated for 30 min at 37°C before transformation of BL21 competent E. *coli* (NEB) cells. Plasmid sequences were confirmed through sanger sequences of the insert using T7 and T7-term primers from Azenta.

Trimerization domains were ordered as eblock fragments (IDT) with a ccdb selective marker (flanked by BsaI cut sites) either at the 5’ or 3’ of the trimerization domain and then inserted into a modified pET29b+ vector using PaqCI (NEB). Briefly, 2:1 molar ratio of trimerization domain:plasmid was ligated at 37°C for 20 min in a 10 μl mixture reaction containing: 1 μl T4 ligase buffer (NEB), 0.5 μl PaqCI (NEB), 0.5 μl T4 DNA ligase (NEB) and 0.25 μl PaqCI activator (NEB). Ligation reactions were then transformed into NEB-stable competent E. *coli* cells. After verifying the sequences of these plasmids, an eblock fragment encoding *E. coli* codon optimized cb3 was inserted into the pET29b+ containing the trimerization domains with the same golden gate assembly reaction described above.

#### MERS-CoV RBD monovalent and multivalent miniproteins design

The design of cb3 followed the same procedure as the binders designed against arbitrary targets of interest^25,32^. First, a library of 30,000 helical bundles were docked onto the DPP4 interface focusing on W553, L506, V555 and Y540 on the MERS-CoV RBD from PDB 4KR0^15^ using Rifgen-Patchdock-Rifdock, which identifies amino acid side chains that form favourable interactions with the target interface and then docks predicted miniprotein structures to determine which can accurately scaffold those interactions^31,32,51^. Rifdock outputs were then sorted using the predicted metrics after a minimal sequence design procedure with the best scoring designs from this short FastDesign implementation taken forward to full sequence design, a process described in depth previously^32^. After full sequence design for a subset of the best scoring docks, these designs were ranked by contact molecular surface, rosetta ΔΔg and contact molecular surface to critical hydrophobic residues. Interface secondary structural elements were extracted from the best designs, clustered, and ranked by ΔΔG to provide 3,000 *de novo* interface motifs. The 30,000 helical bundles were superimposed on these interface motifs to generate more docks which were subjected to the same design and ranking process as the Rifdock outputs. These motif-grafted outputs were combined with the Rifdock outputs and the best designs from the union ordered.

Trimer1 trimerization domain is a Rosetta-based design previously described^24^. Trimers 2–5 were developed using AlphaFold2 MCMC hallucination followed by ProteinMPNN sequence design^37,52^. Trimer 6 was designed using AlphaFold2 MCMC hallucination followed by sequence design using the Rosetta Fast Design function using a position specific scoring matrix generated from 9-mer fragments extracted from the PDB^53,54^, and layer design limiting amino acids on surface exposed loops and strands to the following amino acids: DEGHKNPRQST.

#### Recombinant protein production

MERS-CoV-RBD and MERS-CoV-2P S were expressed in Expi293F cells at 37°C and 8 % CO_2_. 100 ml of cells were transfected with 640 μl of Expifectamine and 100 μg of the respective plasmids, following the manufacturer’s indications. Four days post-transfection, supernatants were clarified by centrifugation at 3500 rpm for 15 min, supplemented with 300 mM NaCl and 25 mM sodium phosphate pH 7.4, bound to HisPur Cobalt Resin previously equilibrated with binding buffer (25 mM sodium phosphate pH 7.4 and 300 mM NaCl), and mixed on an end-over-end rotator at 4°C for 1 h. Recombinant proteins were eluted using 25 mM sodium phosphate pH 7.4, 300 mM NaCl, and 500 mM imidazole. Proteins were further purified by size-exclusion chromatography using a Superdex 75 Increase 10/300 GL column pre-equilibrated in 25 mM Tris-HCl pH 8.0, 150 mM NaCl. Ectodomain of human DPP4 fused to an FC-tag was expressed following a similar protocol. After 4 days of expression, supernatants were clarified and passed through a HiTrap Protein A HP column (Cytiva) previously equilibrated in 20 mM sodium phosphate pH 8.0. Proteins were eluted using 0.1 M citric acid pH 3.0 in individual tubes (1 ml final volume per fraction) containing 200 μl of 1 M Tris-HCl pH 9.0 to immediately neutralize the low pH needed for elution. Fractions containing purified desired proteins were pooled and buffer exchanged to 25 mM Tris-HCl pH 8.0, 150 mM NaCl.

Miniprotein monomers and trimers were expressed from glycerol stocks inoculated into 50 ml of autoinduction media and grown at 37°C for 18 h. Cells were harvested via centrifugation at 4000 x g for 5 min followed by resuspension in 5 ml lysis buffer (20 mM Tris-HCl pH 8.0, 300 mM NaCl, 25 mM imidazole, 1 mM PMSF, 0.1 mg/ml lysozyme, 10 μg/ml DNAse I). Cells were lysed by sonication and then centrifuged at 13000 x g for 10 min to clarify the lysates before loading on to 0.5 ml Ni-NTA resin (Thermo Fisher). The resin was washed with 3 × 5 column volumes (CVs) of 20 mM Tris-HCl pH 8.0, 300 mM NaCl, 25 mM imidazole and then eluted in 20 mM Tris-HCl pH 8.0, 300 mM NaCl, 500 mM imidazole. Proteins were further purified via size exclusion chromatography (SEC) into TBS (20 mM Tris-HCl pH 7.4, 150 mM NaCl) on a Superdex 75 10–300 GL Increase column for monomers and Superdex 200 10–300 GL Increase column for trimers.

For neutralization and *in vivo* studies, miniproteins were prepared with reduced endotoxin through the addition of four detergent washes while bound to the Ni-NTA resin (Thermo Fisher) using TBS with 0.75% CHAPS. For each wash, the resin was incubated at 37°C for 15 min prior to allowing the buffer to flow through. Subsequently, the resin was washed with 3 × 10 CVs of wash buffer to remove remaining detergent before elution. Prior to SEC, column and AKTA were soaked for 4 h in 0.5 M in NaOH before being washed with TBS containing 0.75% CHAPS.

For crystallography, tagless cb3 and HALC3_919 were produced through cleavage at the SNAC tag at the C-terminus of the design. Briefly, cb3- and Trimer6-SNAC tagged were first bound to Ni-NTA resin (Thermo Fisher) and subsequently incubated in 0.1M CHES, 0.1M NaCl, 0.1 M acetone oxime, 0.5 M guanidinium HCl and 2 mM NiCl_2_ for 24 h prior to collecting the tagless proteins from the flow through. Proteins were further purified by size exclusion chromatography as described above.

#### Binding assays to biotinylated MERS-CoV RBD *via* yeast-surface display

Yeast display experiments were performed as described previously^32^. 25,000 designs were reverse translated, and codon optimized for *S. cerevisiae* expression using DNAworks 2.0^55^ and then ordered as 240 bp DNA oligos (Agilent) with 5’ GGTGGATCAGGAGGTTCG and 3’ GGAAGCGGTGGAAGTGGG adapters. The SSM libraries were ordered in the same manner. Affinity enhancing mutations identified in the SSM libraries were then ordered as DNA ultramers (IDT) with library diversities of approximately 10^6^.

The oligo pools were amplified using Kapa Hifi polymerase (Kapa Biosystems). qPCR reactions were prepared in duplicate 25 μl reactions with the first being run to determine the cycle number for half maximal amplification followed by a second production run. These were then purified from an agarose gel (Zymo Research) and amplified again to obtain sufficient material for transformation. 2 μg of linearized petcon3 and 6 μg of insert were transformed into EBY100 cells (Yeast Resource Center, University of Washington) through electroporation^56,57^.

Yeast cultures were maintained in C-Trp-Ura media (Yeast Resource Center, University of Washington) with 2 % glucose grown at 30°C. Expression was induced by inoculating 10 ml of SGCAA (Yeast Resource Center, University of Washington) with 250 μl of C-Trp-Ura culture. Transformed libraries underwent four sorts. For all sorts, SGCAA-induced cultures were harvested by centrifugation at 4000 x g for 4 min after which they were washed once with PBS supplemented with 1 % bovine serum albumin (BSA). In the first round, cells were stained with only 1% anti-myc fluorescein isothiocyanate (FITC) (Immunology Consultants Laboratory) for 15 min at room temperature followed by three washes in ice cold and resuspended in 50 μl of PBS supplemented with 1 % BSA. FITC positive cells were collected indicating successful design expression based on anti-myc FITC binding to a myc tag at the C-terminus of the design. In the second sort, cells were stained in 1 % anti-myc FITC, 1 μM biotinylated MERS-CoV S RBD and 250 nM of streptavidin-phycoerythrin (SAPE) (Thermo Fisher) for 1 h at room temperature followed by three washes in ice cold PBS supplemented with 1 % BSA. Cells were kept on ice before being resuspended in 50 μl of immediately prior sorting. Cells that were FITC and PE positive were collected. These cells were grown in C-Trp-Ura for two days and then induced before a second sort following the same protocol. A final titration sort at 1000 nM, 200 nM and 40 nM with RBD and SAPE in a 1:1 ratio was performed to identify the highest affinity binders, following the same wash steps as described above. Yeast surface display data and determination of the target concentration that achieves 50 % of the saturating binding signal on yeast was done using published tools^32^.

#### Protein biotinylation

MERS-CoV RBD domain used for BLI and yeast display experiments was biotinylated using BirA biotin-protein ligase standard reaction kit (Avidity) following manufacturer’s protocol. In a typical reaction, 40 mM of B domains were incubated overnight at 4 C with 2.5 mg of BirA enzyme in reaction mixtures containing 1X BiomixB, 1X BiomixA and 40 mM BIO200. RBDs were further separated from BirA by SEC using Superdex 75 increase 10/300 GL (GE LifeSciences) and concentrated using 10 kDa filters (Amicon).

#### Lyophilization

nTrimer1 was buffer exchanged into water and then lyophilized overnight at a concentration of 2 mg/ml using a SP Scientific BenchTop Pro lyophilizer. Lyophilized trimeric miniprotein was stored at room temperature until reconstitution in TBS.

#### Measurement of binding affinities using surface plasmon resonance (SPR)

SPR experiments were performed using a Biacore 8K and the biotin CAP chip (Cytiva) for both the monomeric and trimeric designs. For the monomers, approximately 250 RU of biotinylated MERS-CoV S RBD was captured. An 8-step two-fold single cycle kinetic dilution series from 500 nM was used to assess affinity with 120 s association and 900 s dissociation at 30 ul/min at 25°C. For trimeric constructs, approximately 300 response units of biotinylated MERS-CoV S protein was captured and an 8-step two-fold single cycle kinetic dilution series from 8.3 nM was used to assess affinity with 120 s association and 30 min dissociation at 30 μl/min at 25°C. The chip was regenerated between runs with 0.25 M NaOH and 6 M guanidinium hydrochloride. K_D_s were determined through global langmuir 1:1 model fitting using the Biacore Evaluation software.

#### Crystallization and structure determination

Freshly purified MERS-CoV S RBD was concentrated to 20 mg/ml, mixed with a 2-fold molar excess of purified miniprotein cb3 and incubated ON at 4 before setting up crystallization screening using a mosquito robot. Crystals were obtained at 22°C by hanging drop vapor diffusion when MERS-CoV-RBD/cb3 miniprotein complex was mixed with mother liquor solution (200 nl final volume) containing: 0.16 M MgCl_2_, 0.08 M Tris-HCl pH 8.5, 24 % (w/v) PEG_4000_, 20 % (v/v) glycerol. Crystals were flash frozen in liquid nitrogen using mother liquor supplemented with 20 % glycerol as cryoprotectant. Diffraction data were collected at the National Synchrotron Light Source II (Brookhaven) beamline AMX (17-ID-1), and processed with the XDS software package^58^ in space group P2_1_2_1_2_1_. Initial phases were obtained by molecular replacement using Phenix-Phaser^59^ and the PDB 4L3N^60^ as a search model. Several subsequent rounds of model building, and refinement were performed using Coot^61^ and Phenix-Refine^62^ to arrive at a final model at 1.85 Å resolution. Model validation was done using Molprobity^63^ and Privateer^64^.

#### VSV pseudotyped virus production

Our panel included the S glycoprotein of the following MERS-CoV isolates: EMC/2012 (NC_019843.3EMC/2012), United Kingdom/2012 (NC_038294.1), Jordan/2012 (AHY21469.1), Seoul/2015 (KT374056.1), and Kenya/2019 (OK094446.1). MERS-CoV S pseudotyped vesicular stomatitis virus (VSV) corresponding to the different strains were generated as previously described^65^. Briefly, HEK293T cells seeded in poly-D-lysine coated 10-cm dishes in DMEM supplemented with 10 % FBS and 1 % PenStrep were transfected with a mixture of 24 μg of the corresponding plasmids, 60 μl Lipofectamine 2000 (Life Technologies) in 3 ml of Opti-MEM, following manufacturer’s instructions. After 5 h at 37°C, DMEM supplemented with 20% FBS and 2% PenStrep was added. The next day, cells were washed three times with DMEM and were transduced with VSV ΔG-luc ^66^. After 2 h, virus inoculum was removed and cells were washed five times with DMEM prior to the addition of DMEM supplemented with anti-VSV-G antibody [Il-mouse hybridoma supernatant diluted 1 to 25 (v/v), from CRL-2700, ATCC] to minimize parental background. After 18–24 h, supernatants containing pseudotyped VSV were harvested, centrifuged at 2,000 x g for 5 min to remove cellular debris, filtered with a 0,45 μm membrane, concentrated 10 times using a 30 kDa cut off membrane (Amicon), aliquoted, and frozen at −80°C.

#### Pseudotyped VSV neutralizations

VeroE6-TMPRSS2 cells were seeded into coated clear bottom white walled 96-well plates at 40,000 cells/well and cultured overnight at 37°C. Eleven 2-fold or 3-fold serial dilutions of the corresponding miniprotein were prepared in DMEM. MERS-CoV S pseudotyped VSV diluted 1 to 20 in DMEM were added 1:1 (v/v) to each miniprotein dilution and mixtures of 50 μl volume were incubated for 45–60 min at 37°C. VeroE6-TMPRSS2 cells were washed three times with DMEM and 40 μl of the mixture containing pseudotyped virus and miniprotein were added. Two hours later, 40 μl of DMEM were added to the cells. After 17–20 h, 70 μl of One-Glo-EX substrate (Promega) were added to each well and incubated on a plate shaker in the dark at 37°C. After 5–15 min incubation, plates were read on a Biotek plate reader. Measurements were done in duplicate or triplicate with at least two biological replicates. Relative luciferase units were plotted and normalized in Prism (GraphPad): cells without pseudotyped virus added were defined as 0 % infection or 100 % neutralization, and cells with virus only (no miniprotein) were defined as 100 % infection or 0 % neutralization.

#### Western Blot

Pseudotyped VSV particles (15 μl) were mixed with 4X SDS-PAGE loading buffer, run on a 4%–15% gradient Tris-Glycine Gel (BioRad) and transferred to a PVDF membrane using the protocol “mix molecular weight” of the Trans-Blot Turbo System (BioRad). Membranes were blocked with 5 % milk in TBS-T (20 mM Tris-HCl pH 8.0, 150 mM NaCl supplemented with 0.05 % Tween-20) at room temperature and with agitation. After 1 h, the stem-helix specific antibody B6 monoclonal antibody^49^ was added at 3 μg/ml and incubated overnight at 4°C with agitation. Membranes were washed three times with TBS-T and an Alexa Fluor 680-conjugated goat anti-human secondary antibody (1:50,000 dilution, Jackson ImmunoResearch, 109–625-098) was added and incubated during 1 h at room temperature. Membranes were washed three times with TBS-T after which a LI-COR processor was used to develop the western blot.

#### Biolayer interferometry assay (BLI)

Competition binding experiments were performed using BLI on an Octet Red (Sartorius) instrument operated at 30°C with 1000 rpm shaking. Biotinylated MERS-CoV S RBD at 3 μg/ml was immobilized to reach 1 nm shift in 10X kinetics buffer (Pall) to streptavidin (SA) biosensors (Sartorius) that were pre-hydrated in 10X kinetics buffer for 10 min prior the experiment. Biosensors were subsequently dipped into 10x Kinetics buffer to stabilize and remove any unbound protein after which loaded biosensors were dipped into a solution containing either nTrimer1 at 0.5 nM in 10X kinetics buffer or only 10X kinetics buffer, until steady state was achieved (1^st^ association step). A 2^nd^ association step was followed by dipping biosensors in a solution containing 0.5 μM nTrimer1 and 0.5 μM hDPP4 or only 0.5 μM hDPP4 for 300 seconds. Dissociation phase was followed by dipping the tips into the 10X Kinetics buffer. The data were plotted in Graphpad Prism (v.10.2.2).

#### Spike-mediated cell-cell fusion assay

We use a split GFP system to study whether spike mediated-cell fusion is inhibited by mini proteins. Experiments were conducted as previously described^67^ with some modifications. In brief, BHK-21-GFP_1–10_ cells were seeded into 6-well plates at a density of 1 × 10^6^ cells per well. The following day, the growth media was removed, cells were washed one time with DMEM, placed in DMEM containing 10% FBS and 1% PenStrep and transfected with 4 μg per well of the corresponding S protein using Lipofectamine 2000. The same day, VeroE6-TMPRSS2-GFP_11_ cells were split into 96-well, glass bottom, black walled plates (CellVis) at a density of 36,000 cells per well. Twenty-four hours after transfection, BHK-21-GFP_1–10_ expressing the S protein were washed three times using FluoroBrite DMEM (Thermo Fisher), detached using an enzyme-free cell dissociation buffer (Gibco), passed through a cell strainer to remove aggregates and incubated at 65,000 cells/ml (for MERS-CoV S Jordan/2012 and United Kingdom/2012, or at 90,000 cells/ml (for EMC/2012, Seoul/2015 and Kenya/2019) with different concentrations of miniprotein nTrimer1 during 30–45 min at 37°C. VeroE6-TMPRSS2-GFP_11_ cells were washed four times with FluoroBrite DMEM and the transfected BHK-21-GFP_1–10_ cells incubated with the miniprotein were plated on top of it. Cells were incubated at 37°C and 5 % CO_2_ in a Cytation 7 plate Imager (Biotek) and both brightfield and GFP images were collected every 30 min for 18 h. Fusogenicity was assessed by measuring the area showing GFP fluorescence for each image using Gen5 Image Prime v3.11 software.

#### CryoEM sample preparation, data collection and data processing for MERS-CoV S in complex with nTrimer1 and nTrimer1_linker 7

Complexes between MERS-CoV S at 1. 5 mg/ml and nTrimer1 or nTrimer1_linker 7 were formed by adding a 3-fold molar excess of the miniprotein and incubating 5–10 min at 4°C. 3μl of MERS-CoV S in complex with nTrimer1 were diluted 1 to 10 and loaded at 0.15 mg/ml onto freshly glow-discharged NiTi grids covered with a thin layer of home-made continuous carbon prior to plunge freezing using a vitrobot MarkIV (ThermoFisher Scientific) with a blot force of −1 and 4.5 sec blot time at 100% humidity and 21°C. For MERS-CoV S in complex with nTrimer1_linker 7, 3 μl of S of undiluted complex at 1.5 mg/ml were loaded onto UltrAufoil^68^ grids prior to plunge freezing using a vitrobot MarkIV with a blot force of 0 and 6–6.5 sec blot time at 100% humidity and 21°C. For MERS-CoV S in complexes with nTrimer1 and nTrimer1_linker 7, 13,530 and 8751 movies were collected, respectively, with a defocus range between −0.8 and −2.0 μm. Both datasets were acquired using a FEI Titan Krios transmission electron microscope operated at 300 kV equipped with a Gatan K3 direct detector and a Gatan Quantum GIF energy filter, operated with a slit width of 20eV. Automated data collection was carried out using the Leginon software^69^ at a nominal magnification of 105,000x corresponding to a pixel size of 0.843 A°. The dose rate was adjusted to 9 counts/pixel/s, and each movie was acquired in counting mode fractionated in 100 frames of 40 ms. Movie frame alignment, estimation of the microscope contrast-transfer function parameters, particle picking and extraction were carried out using Warp^70^. One round of reference-free 2D classification was performed using cryoSPARC^71^ with binned particles to select well-defined particle images. To further improve particle picking, we trained the Topaz picker^72^ on the Warp-picked particles belonging to the selected 2D classes. Topaz-picked particles were extracted and 2D classified using cryoSPARC. Topaz-duplicated picked particles were removed using 70 Å (for MERS-CoV S-nTrimer1 complex) or 90 Å (for MERS-CoV S-nTrimer1_linker 7 complex) as a minimum distance cutoff. Initial model was generated using ab-initio reconstruction in cryoSPARC and used as reference for a non-uniform refinement^73^ (NUR). Particles were transferred from cryoSPARC to Relion^74–76^ using pyem^77^ (https://github.com/asarnow/pyem) to be subjected to one round of 3D classification with 50 iterations, using the NUR map as a reference model (angular sampling 7.5 ° for 25 iterations and 1.8 ° with local search for 25 iterations) and without imposing symmetry. For MERS-CoV S in complex with nTrimer1, we identified two main populations corresponding to trimers with two or with 3 RBDs engaged with the bound miniprotein. Each of the two populations were processed separately after this step. Particles selected were then subjected to NUR with per-particle defocus refinement using cryoSPARC applying C3 symmetry for particles with 3 RBDs engaged by nTrimer1 and without symmetry for particles engaging 2 RBDs before carrying out Bayesian polishing^78^ using Relion. For MERS-CoV S in complex with nTrimer1_linker 7, selected particles from Relion 3D classification were subjected to another round of 2D classification followed by a Bayesian polishing^78^ using Relion. During Bayesian polishing^78^ particles were re-extracted with a box size of 512 pixels at a pixel size of 1 A°. After polishing, particles from both complexes were subjected to 2D classification in cryoSPARC followed by either NUR with per-particle defocus refinement for MERS-CoV S in complex with nTrimer1 or ab-initio and heterogenous refinement to further clean the dataset before NUR with per-particle defocus refinement for MERS-CoV S in complex with nTrimer1_linker 7. To improve the density of the MERS-CoV S RBD in complex with nTrimer1_linker 7 map, particles were subjected to Relion 3D focused classification without refining angles and shifts using a soft mask encompassing one cb3-bound RBD. Selected particles were subjected in paralell i) to a local refinement using a soft mask encompassing two RBDs with bound cb3s and local resolution estimation using CryoSPARC, which yielded a 3.2 Å resolution map; and ii) to a Relion 3D focused classification without refining angles and shifts using a soft mask encompassing the most weakly resolved RBD and cb3 region (that was not part of the mask used for the first focused 3D classification) followed by NUR with per-particle defocus refinement using the selected particles, that yielded a global map in which each RBDs were engaged by one cb3. Reported resolutions are based on the gold-standard Fourier shell correlation using 0.143 criterion^79^ and Fourier shell correlation curves were corrected for the effects of soft masking by high-resolution noise substitution^80^.

#### CryoEM model building and refinement

PDB 4L3N was used as an initial model and Coot^61^ was used to manually build the model. Model was refined and rebuilt into the maps using Coot and Rosetta^81^. Model validation was done using Molprobity^63^ and Privateer^64^. Figures were generated using UCSF ChimeraX^82^.

#### *In vivo* experiments

Mice were inoculated with either vehicle (TBS), nTrimer1, influenza miniprotein (Flu-HA) monomer^32^ or were left untreated. The Flu-Ha miniprotein, MSGSQHDEFGKWMIEKLKEAVDRGNKISAEFLYNLGKNFVRNPDILKQMEELRKSLHGSGSHHWGSTHHHHHH, was redesigned to improve its solubility. Immediately prior to dosing or infection, mice were anesthetized with a mixture of xylazine and ketamine prior to nTrimer1 treatment (6.25 mg/kg) and viral infection with mouse-adapted MERS-CoV-m35c4 (1×10^5^ PFU) via intranasal instillation.. Mice were monitored daily for weight loss and signs of morbidity. At the indicated time post infection, mice were euthanized via isoflurane overdose. Lung tissue was assessed for gross pathology for signs of hemorrhage using a semi-quantitative 0–4 scale (congestion score), where 0 is a normal pink healthy lung and 4 is a completely dark red lung^83^. The large lobe was harvested for histopathology and processed. The inferior lobe was harvested in PBS with glass beads and stored at −80°C for viral titration by plaque assay.

#### Lung histopathology

Tissue sections were prepared as previously described^84^ from the left lung lobe and were microscopically examined for features of virus-associated histopathology by a board-certified veterinary pathologist without knowledge of individual animal infection or treatment (“blinded analysis”).

For MERS-CoV antigen staining, paraffin-embedded sections were baked at 60°C for 2–4 hours, deparaffinized in xylene and graded ethanol, rehydrated, and subjected to antigen retrieval in 0.1 M sodium citrate (pH 6.0) with three microwave cycles (100% power for 6.5 min, then 60% power for 6 min twice). After cooling and rinsing, slides were blocked (Blocking One Histo, 20 min, RT), then incubated overnight at 4°C with a MERS nucleocapsid antibody (polyclonal mouse serum, 1:500) in Blocking One Histo (1:20 in PBST). After PBST washes, a species-specific secondary antibody was applied for 60 min at room temperature..For caspase 3 staining,formalin fixed paraffin embedded samples were sectioned at 4 – 5 microns and placed on positively charged slides. Slides were deparaffinized followed by heat induced epitope retrieval utilizing Citrate pH 6.0 buffer (Scytek Labs – Logan, UT) in a vegetable steamer, followed by endogenous peroxidase blocking in hydrogen peroxide + methanol for 30 min; running tap and distilled water rinses. The following protocol was performed on the Biocare intelliPath Flex^™^ (Biocare Medical – Pacheco, Ca) automated immunostaining platform using ProMARK^™^ detection reagents with AutoWash buffer rinses between each step. Non-Specific Proteins were blocked with Rodent Block M (Biocare) for 20 min followed by incubation in Rabbit anti-Caspase 3 Primary antibody (Cell Signaling – Danvers, MA) diluted 1:50 in normal antibody diluent (Syctek) overnight at 4°C; then Rabbit on Rodent HRP Polymer (Biocare) – 30 min. The reaction was developed with AEC (Biocare) – 5 min followed by counterstain with CATHE hematoxylin (Biocare) diluted 1:10 in distilled water for 5 min. Post staining slides are rinsed in distilled water, dehydrated in ethanol, cleared in Xylene and cover slipped with Optic Mount 1 media (Mercedes Medical – Lakewood Ranch, FL).

#### Live-virus neutralization assays

Huh7.5 cells^85^ were seeded in black bottom 96-well plates at 25,000 cells/well and cultured overnight at 37°C. Eight 3-fold serial dilutions of the corresponding miniprotein were prepared in DMEM (5 % FBS). MERS-CoV EMC/2012 expressing nanoluciferase (nLuc) was added 1:1 (v/v) to each miniprotein dilution and incubated for 1 h at 37°C before inoculation onto cells for a final concentration of 800 plaque forming units per well. After 24 h, 25 μl of Nano-Glo substrate (Promega) was added to each well and incubated at room temperature for 3 min before being read on a Promega plate reader. Measurements were done in duplicate. Relative luciferase units were plotted and normalized in Prism (GraphPad) as described above.

### QUANTIFICATION AND STATISTICAL ANALYSIS

As described above, binding K_D_s were determined through global langmuir 1:1 model fitting using the Biacore Evaluation software. All other statistical analyses for neutralization and *in vivo* studies were done using Prism 10. Where applicable, means across independent replicates are reported with error bars indicating the standard error of the mean. Details of tests for statistical significance are specified in the figure legends.

## Supplementary Material

SuppMaterial

FigureS1 **Fig S1. Inhibition of MERS-CoV S-mediated entry into VeroE6-TMPRSS2 cells by monomeric and trimeric designed miniproteins, related to** Figure 1B. **A**, Western blot analysis of VSV pseudotyped particles harboring MERS-CoV EMC/2012, Jordan/2012 or United Kingdom/2012 S detected using the B6 stem-helix monoclonal antibody^49^ as a primary antibody. Full-length S and S_2_ subunit bands are indicated on the right-hand side of the blot. **B-D**, Concentration-dependent inhibition of MERS-CoV S pseudovirus entry into VeroE6-TMPRSS2 cells for MERS-CoV S EMC/2012 (**B**), Jordan/2012 (**C**) and United Kingdom/2012 (**D**) by monomeric miniproteins. **E-F**, MERS-CoV EMC/2012 (**E**), Jordan/2012 (**F**) and United Kingdom/2012 (**G**) S VSV pseudovirus-mediated entry in the presence of various dilutions of the indicated trimeric miniproteins. Monomeric miniprotein cb3 was used as a reference and cb4 as negative control of neutralization. Exp. 1 and Exp. 2 correspond to two biological experiments performed with two different preparations of pseudotyped viruses and miniproteins. Error bars represent the standard error of the mean (SEM) of the technical duplicates. Fits are shown only when neutralization is detected.

FigureS2 **Fig S2. Inhibition of VSV-pseudotyped MERS-CoV S-mediated entry by nTrimer1, related to** Figure 1B and Table 1. **A.** Western blot analysis of VSV pseudotyped particles harboring the indicated MERS-CoV S variants detected using the stem-helix monoclonal antibody B6^49^ as a primary antibody. Mw, molecular weight ladder. Full-length S and S_2_ subunit bands are indicated on the right-hand side of the blot. **B.** MERS-CoV EMC2012, Jordan/2012, United Kingdom/2012, Kenya/2019 and Seoul/2015 S VSV pseudovirus entry in the presence of various dilutions of the indicated miniproteins. Exp. 1 and Exp. 2 correspond to two biological experiments performed with two different preparations of pseudotyped viruses and miniproteins. Error bars represent the standard error of the mean (SEM) of technical duplicates. Fits are shown only when neutralization is detected. **C.** MERS-CoV EMC/2012, Jordan/2012, United Kingdom/2012, Kenya/2019 and Seoul/2015 S pseudovirus entry in the presence of various dilutions of nTrimer1 lyophilized and reconstituted or not lyophilized. A single biological experiment with technical duplicates is shown. Error bars represent the standard error of the mean (SEM) of the technical duplicates. IC_50_ values, expressed in nanomolar, obtained from the experiment shown in the top panels.

FigureS3 **Fig S3. CryoEM data processing and validation of the structure of MERS-CoV S in prefusion conformation in complex with nTrimer1 (cb3-GSG-Trimer1), related to** Figure 2E. **A.** Representative electron micrograph. **B.** 2D class averages. Scale bar of the micrograph and the 2D class averages, 100 nm and 100 Å, respectively. **C.** Cryo-EM data processing flowchart. CTF: contrast transfer function. NUR: non uniform refinement. **D.** Gold-standard Fourier shell correlation curves for the global maps with three and two RBDs engaged are shown in black and gray, respectively. The 0.143 cutoff is indicated by a horizontal dotted black line. **E.** Unsharpened map corresponding to prefusion MERS-CoV S in complex with three nTrimer1 colored by local resolution. **F.** Angular distribution plot with all the particles contributing to the map in E. **G.** Unsharpened map corresponding to the prefusion MERS-CoV S in complex with two nTrimer1 colored by local resolution. **H.** Angular distribution plot with all the particles contributing to the map in G.

FigureS4 **Fig S4. Optimization of the linker length between the miniprotein binding domain cb3 and trimer1 A-B, related to** Figure 2. **A.** MERS-CoV EMC/2012, Jordan/2012, United Kingdom/2012, Kenya/2019 and Seoul/2015 S VSV pseudovirus entry into cells in the presence of various dilutions of nTrimer1 with different linkers lengths between cb3 and trimer1. Miniprotein cb4 was used as a negative control. A single biological experiment with two technical replicates is shown. Error bars represent the standard error of the mean (SEM) of the technical duplicates. **B.** Structural overlay between the cryo-EM structure of the MERS-CoV S RBD in complex with nTrimer1 linker 7 and the X-ray structure of the MERS-CoV S RBD in complex with cb3.

FigureS5 **Fig S5. CryoEM data processing and validation of the structure of prefusion MERS-CoV S in complex with nTrimer1_linker 7 (cb3_GGGSGGGS_trimer1), related to** Figure 2. **A.** Representative electron micrograph. **B.** 2D class averages. Scale bar of the micrograph and the 2D class averages, 100 nm and 100 Å, respectively. **C.** Cryo-EM data processing flowchart. CTF: contrast transfer function. NUR: non uniform refinement. **D.** Gold-standard Fourier shell correlation curves for the global maps (black and red) and locally refined map (blue). The 0.143 cutoff is indicated by a horizontal dotted black line. **E.** Unsharpened map corresponding to the 3D reconstruction of MERS-CoV S (in prefusion conformation) in complex with nTrimer1_linker 7 colored by local resolution. **F.** Locally refined sharpened map corresponding to two neighboring MERS-CoV S RBDs each engaging one cb3 from the nTrimer1_linker 7 miniprotein colored by local resolution. **G.** Global unsharpened map for the MERS-CoV-S in complex with nTrimer1_linker 7 miniprotein obtained after focused classification and colored by local resolution. **H, I.** Angular distribution plots corresponding to the maps shown directly above.

StarMethodsTable **Table S1 related to** Figure 1F–G. X-ray crystallography data collection and refinement statistics of the cb3-MERS-CoV RBD complex.
**Table S2.** List of IC_50_ values obtained from the neutralization curves shown in Fig S1. Monomeric miniprotein cb3 was used as a reference to highlight the improved neutralization exhibited by the trimerization of cb3. Monomeric miniprotein cb4 was used as a negative control. The two IC_50_ values for each pseudotyped virus correspond to two distinct biological experiments performed with two batches of pseudovirus and one batch or miniprotein. The “n” and “c” indices refer to the position N- or C-terminus of the miniprotein cb3 relative to the indicated trimerization domain. Limit of detection (LOD) of neutralization is between 5×10^2^-10^3^ nM (see Fig S1).
**Table S3 related to** Figures 1E and 2C. Summary of binding kinetics for monomeric and trimeric miniproteins to the prefusion MERS-CoV S trimer. K_D_ or apparent K_D_ (K_Dapp_ denoted with a star, due to multivalent binding and avidity) values were determined through global langmuir 1:1 model fitting.
**Table S4.** Sequences of miniproteins, trimerization domains, and fusion expression constructs. Each trimerization domain was tested as an N- and C-terminal fusion with respect to the miniprotein cb3. All constructs were expressed with MSG - design - GS - SNAC tag - 6x his, as described in methods. The name in brackets indicates a previously published name for that homotrimer domain.
**Table S5 related to** Figure 2. Cryo-EM data collection and refinement statistics.

## Figures and Tables

**Table 1.** IC_50_ values for the indicated miniproteins.

IC_50_s (nM)										
miniprotein	EMC/2012		Jordan/2012		United Kingdom/2012		Seoul/2015		Kenya/2019	
nTrimer1	1.1	0.9	1	0.8	1.6	1.6	0.7	0.3	1.3	0.3
nTrimer2	0.8	0.7	0.6	0.6	1.3	1.5	0.2	0.4	0.6	0.15
nTrimer5	1.3	1.1	0.9	1.2	1.9	2.3	0.8	0.7	1.1	0.6
cTrimer3	3.6	2.1	3	2.3	LOD	LOD	5.9	7.2	1.6	0.8
cb3	109.5	229	114.4	146.7	703.9	642	252.2	212.6	117.9	97.17
cb4	LOD	LOD	LOD	LOD	LOD	LOD	LOD	LOD	LOD	LOD

Monomeric miniprotein cb3 was used as a reference and monomeric miniprotein cb4 was used as a negative control. The two IC_50_ values correspond to two different biological replicates using two batches of pseudovirus and two batches of miniproteins.

**STAR Methods Table:** 

REAGENT or RESOURCE	SOURCE	IDENTIFIER
**Antibodies**		
B6 monoclonal antibody (anti-stem helix)	Sauer *et al.*^49^	N/A
AF680 conjugated goat anti-human	Jackson ImmunoResearch	109-625-098
JC57-11 monoclonal antibody (anti-RBD)	Tse *et al.*^21^	N/A
Anti-myc FITC	Immunology Consultants Laboratory	cat# CMYC-45F
Anti-cleaved caspase 3 (Asp175)	Cell Signaling Technology	cat# 9661
Anti-MERS-CoV nucleocapsid polyclonal sera	Cockrell *et al.*^42^	N/A
**Bacterial and virus strains**		
BL21 (DE3)	NEB	cat# C2527I
*S. cerivasiae* (EBY100)	Yeast Resource Centre, University of Washington	EBY100
VSV (G*ΔG-luciferase)	Kaname et al.^66^	N/A
MERS-CoV EMC 2012 nanoluciferase reporter virus	This work	N/A
Mouse adapted MERS-CoV EMC 2012 (m35c4)	This work	N/A
**Chemicals, peptides, and recombinant proteins**		
MERS-CoV S RBD	Addetia *et al.* 2024^9^	N/A
MERS-CoV S trimer	Park *et al.*^13^	N/A
MERS-CoV S trimer biotinylated	Acro Bio	cat# SPN-M82E3
Human DPP4	Addetia *et al.* 2024^9^	N/A
HA miniprotein	Cao *et al.*^25^	N/A
Streptavidin-PE	Thermo Fisher	cat# S866
BirA biotin-protein ligase standard reaction kit	Avidity	cat# BirA500
cb3	This work	N/A
cb4	This work	N/A
cb6	This work	N/A
nTrimer1	This work	N/A
nTrimer2	This work	N/A
nTrimer5	This work	N/A
nTrimer6	This work	N/A
cTrimer1	This work	N/A
cTrimer2	This work	N/A
cTrimer3	This work	N/A
cTrimer4	This work	N/A
cTrimer5	This work	N/A
cTrimer6	This work	N/A
nTrimer1 linker 1	This work	N/A
nTrimer1 linker 2	This work	N/A
nTrimer1 linker 3	This work	N/A
nTrimer1 linker 4	This work	N/A
nTrimer1 linker 5	This work	N/A
nTrimer1 linker 6	This work	N/A
nTrimer1 linker 7	This work	N/A
nTrimer1 linker 8	This work	N/A
**Critical commercial assays**		
Biotin capture kit (SPR)	Cytiva	cat# 28920234
Nano-Glo^®^ Luciferase Assay System	Promega	cat# N1130
**Deposited data**		
MERS-CoV RBD	PDB	4L3N
MERS-CoV RBD	PDB	4KR0
**Experimental models: Cell lines**		
HEK293T	ATCC	cat# CRL-3216
VeroE6-TMPRSS2	JCRB-Cell bank	cat# JCRB1918
Expi293F	Thermo Fisher	cat# A14527
VeroE6-TMPRSS2-GFP_11_	Addetia et al., 2023^67^	N/A
BHK-21-GFP_1–10_	Addetia et al., 2023^67^	N/A
Huh7.5	Blight *et al.*^85^	N/A
**Experimental models: Organisms/strains**		
288/330 C57BL/6J mice	Cockrell *et al.*^42^	N/A
**Recombinant DNA**		
pcDNA3.1 (+) MERS-CoV S Jordan/2012 full-length	GenScript	N/A
pcDNA3.1(+) MERS-CoV EMC/2–12 S full-length	Addetia *et al.*, 2024^9^	N/A
pcDNA3.1(+) MERS-CoV United Kingdom/2012 S full-length	Addetia *et al.*, 2024^9^	N/A
pcDNA3.1(+) MERS-CoV Seoul/2015 S full-length	Addetia *et al.*, 2024^9^	N/A
pcDNA3.1(+) MERS-CoV Kenya/2019 S full-length	Addetia *et al.*, 2024^9^	N/A
B6 plasmid	Sauer *et al.*^9^	N/A
LM0627 cb3	This work	N/A
LM0627 cb4	This work	N/A
LM0627 cb6	This work	N/A
pET29b+ nTrimer1	This work	N/A
pET29b+ nTrimer2	This work	N/A
pET29b+ nTrimer5	This work	N/A
pET29b+ nTrimer6	This work	N/A
pET29b+ cTrimer1	This work	N/A
pET29b+ cTrimer2	This work	N/A
pET29b+ cTrimer3	This work	N/A
pET29b+ cTrimer4	This work	N/A
pET29b+ cTrimer5	This work	N/A
pET29b+ cTrimer6	This work	N/A
LM0627 nTrimer1 linker 1	This work	N/A
LM0627 nTrimer1 linker 2	This work	N/A
LM0627 nTrimer1 linker 3	This work	N/A
LM0627 nTrimer1 linker 4	This work	N/A
LM0627 nTrimer1 linker 5	This work	N/A
LM0627 nTrimer1 linker 6	This work	N/A
LM0627 nTrimer1 linker 7	This work	N/A
LM0627 nTrimer1 linker 8	This work	N/A
pETcon3 yeast display design library	This work	N/A
**Software and algorithms**		
Rosetta	RosettaCommons	https://rosettacommons.org/software/
Biacore Evaluation software	Cytiva	https://www.cytivalifesciences.com/en/us/shop/protein-analysis/spr-label-free-analysis/spr-software-and-extensions/biacore-insight-evaluation-software-p-23528
Prism 10	GraphPad	https://www.graphpad.com/
Gen5 image prime v3.11	Agilent	https://www.agilent.com/en/product/microplate-instrumentation/microplate-instrumentation-control-analysis-software/imager-reader-control-analysis-software
PyMol 3	Schrödinger LLC	https://pymol.org/
UCSF Chimera X	Meng *et al.*^82^	https://www.rbvi.ucsf.edu/chimerax/
Molprobity	Williams *et al.*^63^	http://molprobity.biochem.duke.edu/
Privateer	Agirre *et al.*^64^	https://privateer.york.ac.uk/
Phenix-Refine		https://phenix-online.org/documentation/index.html
Phenix-Phaser	McCoy *et al.*^59^	https://phenix-online.org/documentation/index.html
Coot	Emsley *et al.*^61^	https://www2.mrc-lmb.cam.ac.uk/personal/pemsley/coot/
CryoSPARC	Punjani *et al.*^71^	https://cryosparc.com/
AlphaFold2 MCMC hallucination	Wicky *et al.*^37^	https://github.com/bwicky/oligomer_hallucination
ProteinMPNN	Dauparas *et al.*^52^	https://github.com/dauparas/ProteinMPNN
DNAworks 2.0	Hoover *et al.*^55^	https://github.com/davidhoover/DNAWorks

## Highlights

- De novo designed miniprotein cb3 binds to MERS-CoV RBD and blocks DPP4 attachment
- Homotrimer of cb3 neutralizes MERS-CoV S pseudovirus strains and authentic MERS-CoV
- Cryo-EM reveals that nTrimer1 engages multiple MERS-CoV RBDs on the same S
- nTrimer1 protects against MERS-CoV-induced disease in a mouse model
