# Supplementary material for: Designed miniproteins potently inhibit and protect against MERS-CoV
Source: Cell Rep. Author manuscript; Available in PMC 2025 Jul 20. (PMC12276895; doi:10.1016/j.celrep.2025.115760)
Supplement: SuppMaterial [file NIHMS2090671-supplement-SuppMaterial.pdf]

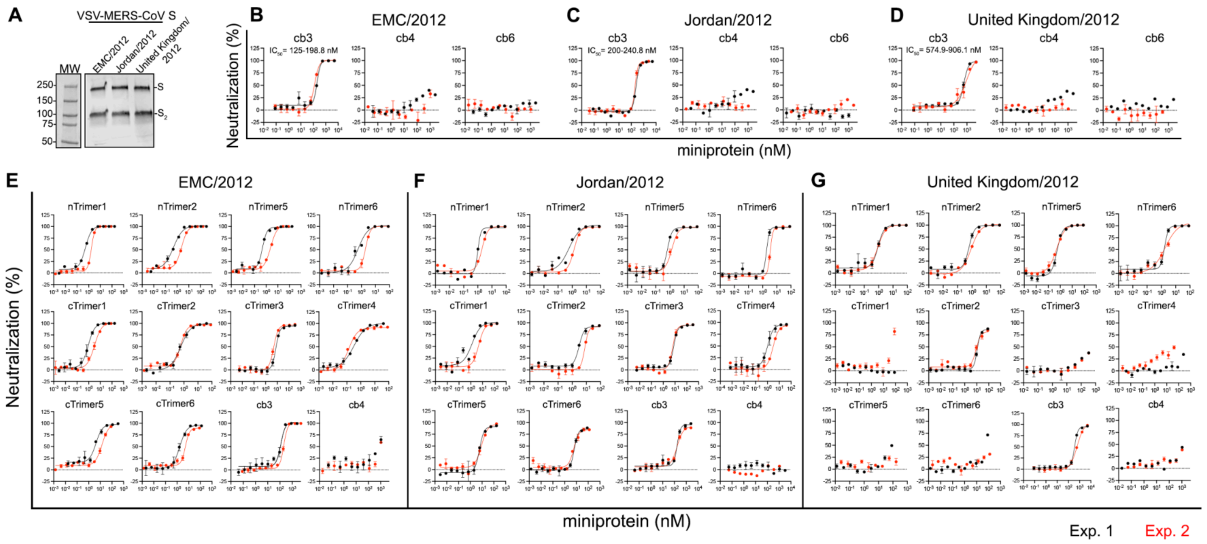

Fig S1. Inhibition of MERS-CoV S-mediated entry into VeroE6-TMPRSS2 cells by monomeric and trimeric designed miniproteins, related to Figure 1B. A, Western blot analysis of VSV pseudotyped particles harboring MERS-CoV EMC/2012, Jordan/2012 or United Kingdom/2012 S detected using the B6 stem-helix monoclonal antibody as a primary antibody. Full-length S and S_2 subunit bands are indicated on the right-hand side of the blot. **B-D**, Concentration-dependent inhibition of MERS-CoV S pseudovirus entry into VeroE6-TMPRSS2 cells for MERS-CoV S EMC/2012 (**B**), Jordan/2012 (**C**) and United Kingdom/2012 (**D**) by monomeric miniproteins. **E-F**, MERS-CoV EMC/2012 (**E**), Jordan/2012 (**F**) and United Kingdom/2012 (**G**) S VSV pseudovirus-mediated entry in the presence of various dilutions of the indicated trimeric miniproteins. Monomeric miniprotein cb3 was used as a reference and cb4 as negative control of neutralization. Exp. 1 and Exp. 2 correspond to two biological experiments performed with two different preparations of pseudotyped viruses and miniproteins. Error bars represent the standard error of the mean (SEM) of the technical duplicates. Fits are shown only when neutralization is detected.

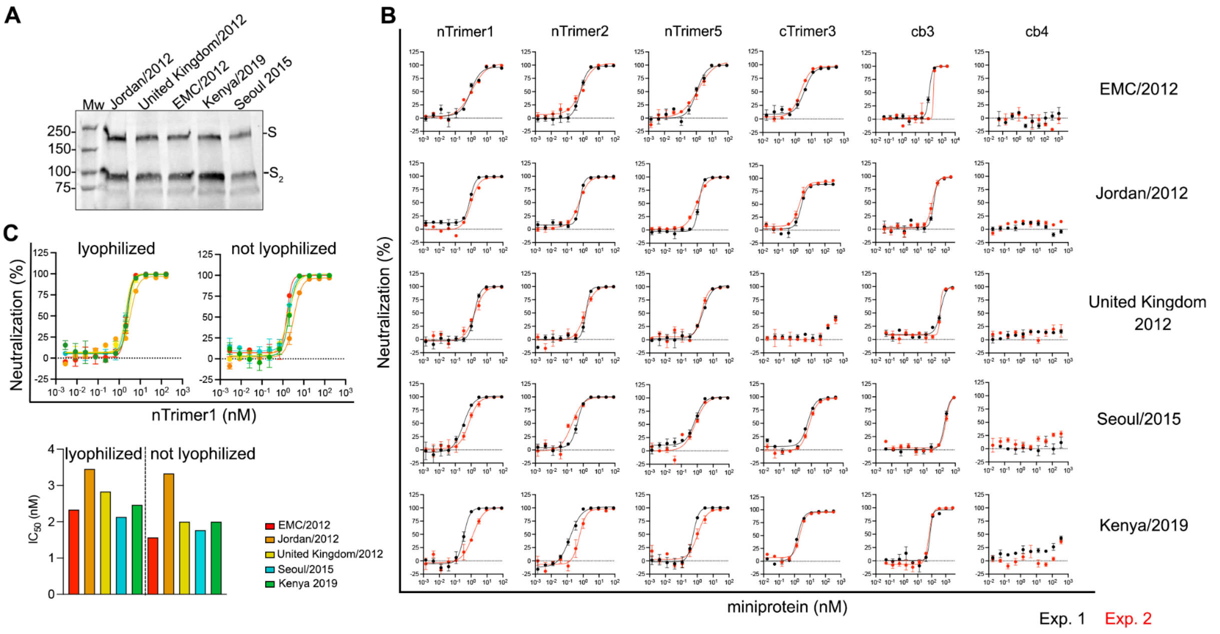

Fig S2. Inhibition of VSV-pseudotyped MERS-CoV S-mediated entry by nTrimer1, related to Figure 1B and Table 1. A. Western blot analysis of VSV pseudotyped particles harboring the indicated MERS-CoV S variants detected using the stem-helix monoclonal antibody B6[49] as a primary antibody. Mw, molecular weight ladder. Full-length S and S_2 subunit bands are indicated on the right-hand side of the blot. **B.** MERS-CoV EMC2012, Jordan/2012, United Kingdom/2012, Kenya/2019 and Seoul/2015 S VSV pseudovirus entry in the presence of various dilutions of the indicated miniproteins. Exp. 1 and Exp. 2 correspond to two biological experiments performed with two different preparations of pseudotyped viruses and miniproteins. Error bars represent the standard error of the mean (SEM) of technical duplicates. Fits are shown only when neutralization is detected. **C**. MERS-CoV EMC/2012, Jordan/2012, United Kingdom/2012, Kenya/2019 and Seoul/2015 S pseudovirus entry in the presence of various dilutions of nTrimer1 lyophilized and reconstituted or not lyophilized. A single biological experiment with technical duplicates is shown. Error bars represent the standard error of the mean (SEM) of the technical duplicates. IC_{50} values, expressed in nanomolar, obtained from the experiment shown in the top panels.

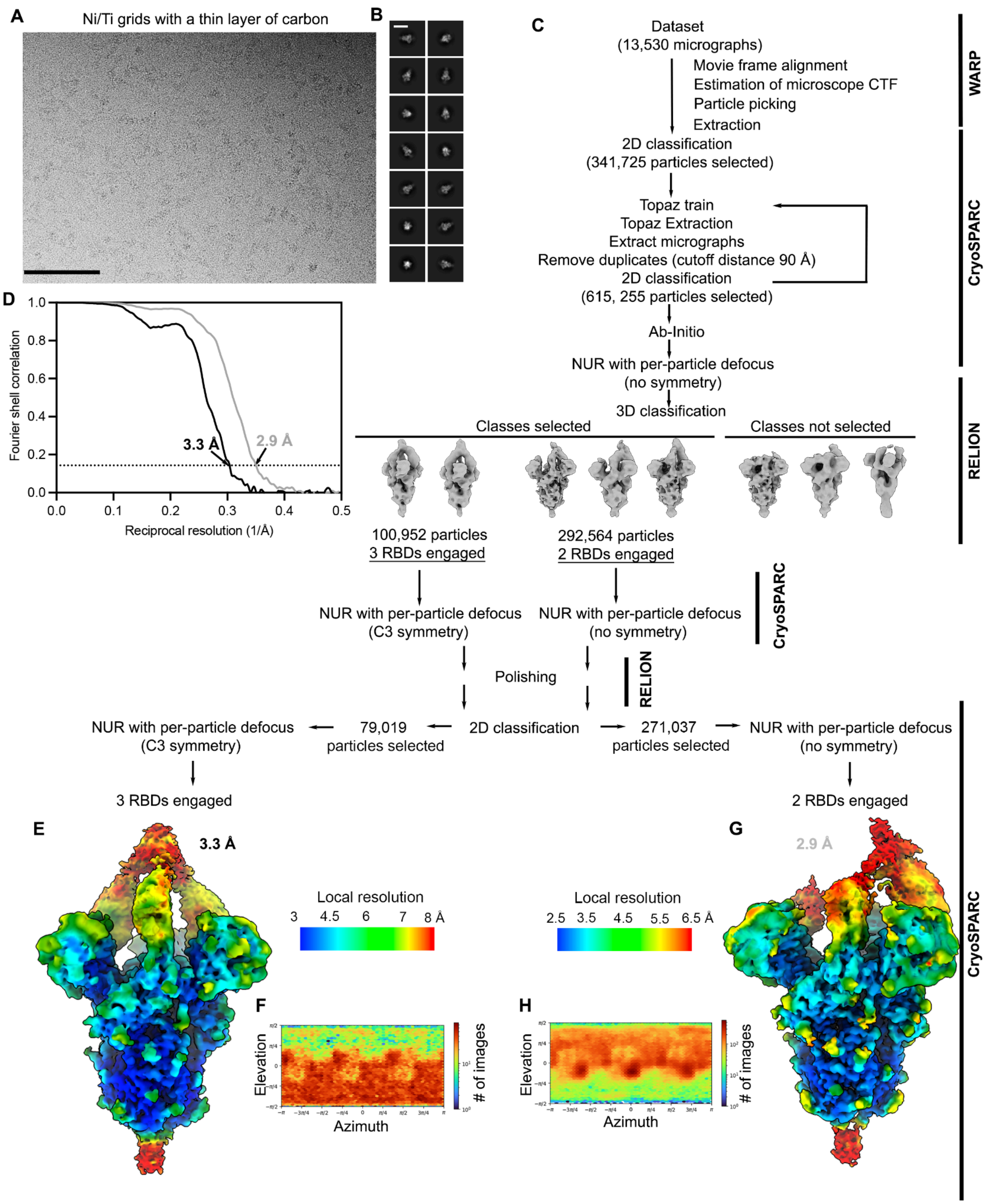

Fig S3. CryoEM data processing and validation of the structure of MERS-CoV S in prefusion conformation in complex with nTrimer1 (cb3-GSG-Trimer1), related to Figure 2E. A. Representative electron micrograph. **B.** 2D class averages. Scale bar of the micrograph and the 2D class averages, 100 nm and 100 Å, respectively. **C.** Cryo-EM data processing flowchart. CTF: contrast transfer function. NUR: non uniform refinement. **D.** Gold-standard Fourier shell correlation curves for the global maps with three and two RBDs engaged are shown in black and gray, respectively. The 0.143 cutoff is indicated by a horizontal dotted black line. **E.** Unsharpened map corresponding to prefusion MERS-CoV S in complex with three nTrimer1 colored by local resolution. **F.** Angular distribution plot with all the particles contributing to the map in E. **G.** Unsharpened map corresponding to the prefusion MERS-CoV S in complex with two nTrimer1 colored by local resolution. **H.** Angular distribution plot with all the particles contributing to the map in G.

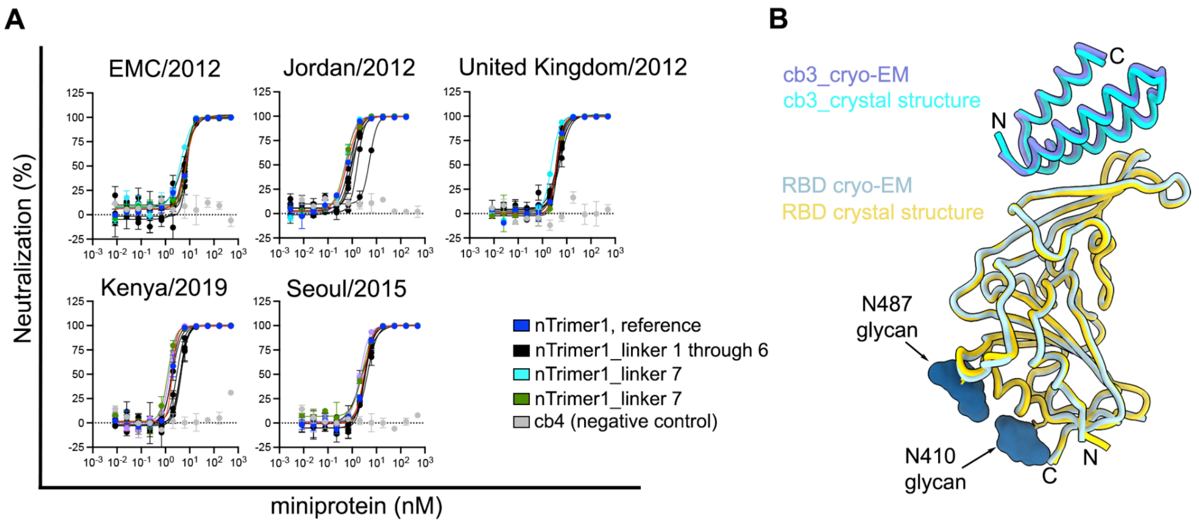

Fig S4. **Optimization of the linker length between the miniprotein binding domain cb3 and trimer1 A-B, related to Figure 2**. **A.** MERS-CoV EMC/2012, Jordan/2012, United Kingdom/2012, Kenya/2019 and Seoul/2015 S VSV pseudovirus entry into cells in the presence of various dilutions of nTrimer1 with different linkers lengths between cb3 and trimer1. Miniprotein cb4 was used as a negative control. A single biological experiment with two technical replicates is shown. Error bars represent the standard error of the mean (SEM) of the technical duplicates. **B.** Structural overlay between the cryo-EM structure of the MERS-CoV S RBD in complex with nTrimer1 linker 7 and the X-ray structure of the MERS-CoV S RBD in complex with cb3.

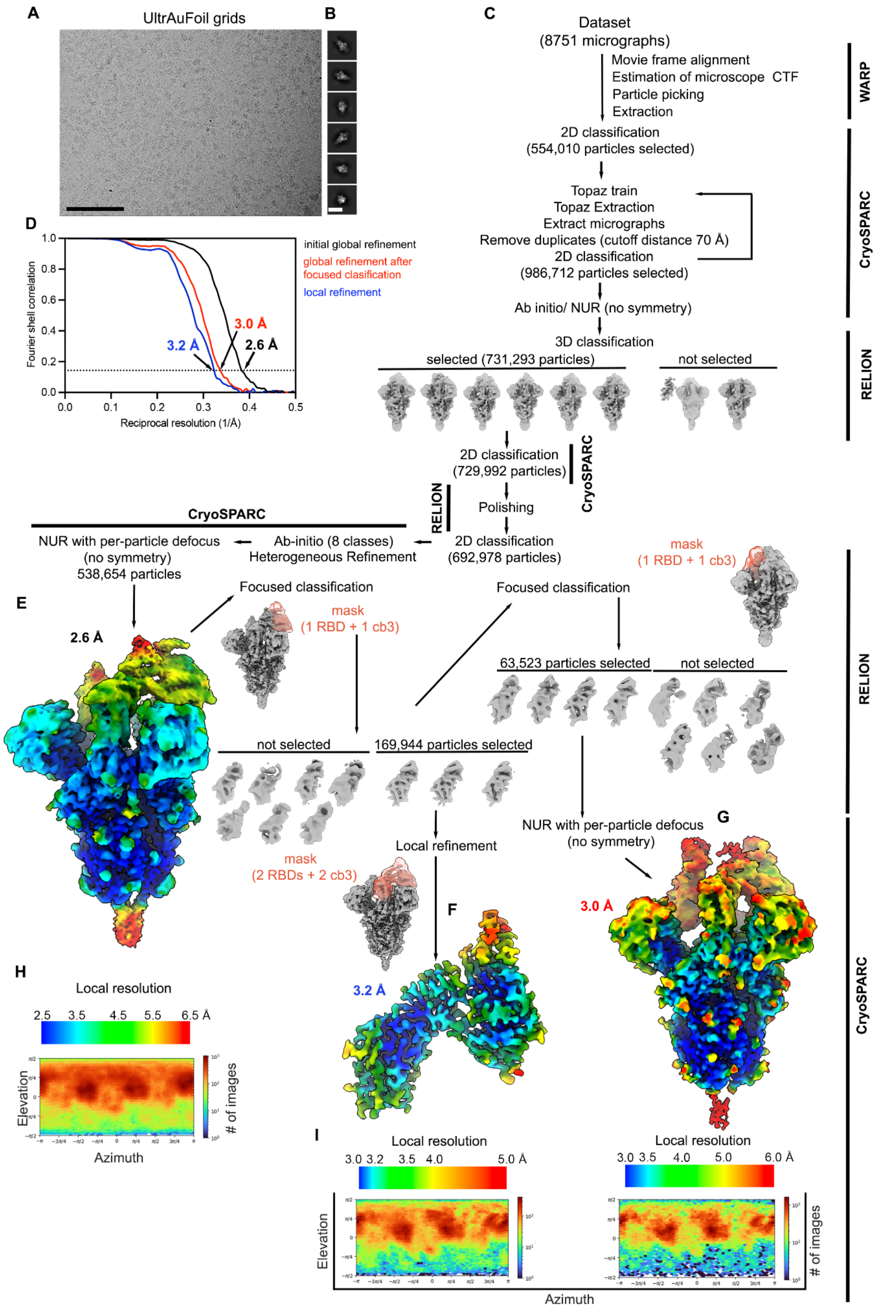

Fig S5 CryoEM data processing and validation of the structure of prefusion MERS-CoV S in complex with nTrimer1_linker 7 (cb3_GGGSGGGS_trimer1), related to Figure 2. A. Representative electron micrograph. **B.** 2D class averages. Scale bar of the micrograph and the 2D class averages, 100 nm and 100 Å, respectively. **C.** Cryo-EM data processing flowchart. CTF: contrast transfer function. NUR: non uniform refinement. **D**. Gold-standard Fourier shell correlation curves for the global maps (black and red) and locally refined map (blue). The 0.143 cutoff is indicated by a horizontal dotted black line. **E.** Unsharpened map corresponding to the 3D reconstruction of MERS-CoV S (in prefusion conformation) in complex with nTrimer1_linker 7 colored by local resolution. **F.** Locally refined sharpened map corresponding to two neighboring MERS-CoV S RBDs each engaging one cb3 from the nTrimer1_linker 7 miniprotein colored by local resolution. **G.** Global unsharpened map for the MERS-CoV-S in complex with nTrimer1_linker 7 miniprotein obtained after focused classification and colored by local resolution. **H, I.** Angular distribution plots corresponding to the maps shown directly above.

Table S1 related to Figure 1F-G. X-ray crystallography data collection and refinement statistics of the cb3-MERS-CoV RBD complex.

	MERS-CoV RBD:cb3
PDB ID	9DGO
Space Group	$P2_12_12_1$
**Cell dimensions**	
a, b, c (Å)	61.619, 76.291, 136.235
α, β, γ (°)	90, 90, 90
Resolution range (Å)	31.55-1.85 (1.95-1.85)
CC1/2	0.999 (0.79)
Rmerge	0.023 (0.378)
I/σ(I)	14.7 (1.9)
Completeness (%)	100 (99.9)
Redundancy	2 (2)
**Refinement**	
No. reflections	55567
Rwork/Rfree	18.96/22.06
Nº of atoms	
Protein	4116
Ligand	
Water	307
B factor (Wilson plot)	33.05
**R.m.s. deviations**	
Bond lengths (Å)	0.007
Bond angles (°)	0.849
Ramachandran favored (%)	99.43
Ramachandran allowed (%)	0.57
Ramachandran outliers (%)	0.0

- Data in parentheses are for the highest resolution shell
- $R_{merge} = \sum(\sum|I_i - \langle I \rangle|/\sum|I|)$, where the first ∑ is the sum over all reflections, and the second ∑ is the sum over all measurements of a given reflection, with I_i being the *i*th measurement of the intensity of the reflection and $\langle I \rangle$ the average intensity of that reflection.
- $R_{work}/R_{free} = \sum(|F_o| - \langle|F_c|\rangle)/\sum|F_o|$, where $\langle|F_c|\rangle$ is the expectation of $|F_c|$ under the probability model used to define the likelihood function. The sum is overall reflections.

Table S2. List of IC_{50} values obtained from the neutralization curves shown in Fig S1. Monomeric miniprotein cb3 was used as a reference to highlight the improved neutralization exhibited by the trimerization of cb3. Monomeric miniprotein cb4 was used as a negative control. The two IC_{50} values for each pseudotyped virus correspond to two distinct biological experiments performed with two batches of pseudovirus and one batch or miniprotein. The “n” and “c” indices refer to the position N- or C-terminus of the miniprotein cb3 relative to the indicated trimerization domain. Limit of detection (LOD) of neutralization is between $5x10^2$-10^3 nM (see Fig S1).

IC50s (nM)						
miniprotein	EMC/2012		Jordan/2012		United Kingdom/2012	
nTrimer1	1.7	0.5	2	1	0.9	0.9
nTrimer2	2.4	0.5	1.3	0.6	0.9	0.6
nTrimer5	2.4	0.5	1.3	0.9	0.5	0.5
nTrimer6	1.9	0.6	3.4	1.9	1.8	1.6
cTrimer1	3.1	0.8	4.7	1.3	0.3	NN
cTrimer2	0.6	0.6	7.8	3.1	8.9	11
cTrimer3	4.9	7.6	6.3	7.7	LOD	LOD
cTrimer4	0.1	0.3	2.9	1	LOD	LOD
cTrimer5	13.7	4.2	8.7	5.9	LOD	LOD
cTrimer6	5.1	1.7	5.9	6.6	LOD	LOD
cb3	200.3	115.8	167.6	169	607.2	355.3
cb4	LOD	LOD	LOD	LOD	LOD	LOD

Table S3 related to Figures 1E and 2C. Summary of binding kinetics for monomeric and trimeric miniproteins to the prefusion MERS-CoV S trimer. K_D or apparent K_D (K_{Dapp} denoted with a star, due to multivalent binding and avidity) values were determined through global langmuir 1:1 model fitting.

**Design**	**k_{on} ($M^{-1}s^{-1}$)**	**k_{off} (s^{-1})**	**K_D (M)**
cb3	3.0E6	1.1E-2	3.7E-9
cb4	3.5E5	2.2E-2	6.1E-8
cb6	4.2E5	1.8E-2	4.4E-8
nTrimer1	2.0E6*	4.2E-5*	2.1E-11*
nTrimer2	2.0E6*	5.3E-5*	2.7E-11*
nTrimer5	1.4E6*	4.7E-5*	3.2E-11*
nTrimer6	1.7E6*	6.0E-5*	3.6E-11*
cTrimer3	1.4E5*	2.6E-3*	1.8E-8*

Table S4. Sequences of miniproteins, trimerization domains, and fusion expression constructs. Each trimerization domain was tested as an N- and C-terminal fusion with respect to the miniprotein cb3. All constructs were expressed with MSG - design - GS - SNAC tag - 6x his, as described in methods. The name in brackets indicates a previously published name for that homotrimer domain.

Design	Sequence
cb3	SPVKRFVREVLEEAEEAYEKGDRRQFEELLWLAEWAARDANDEELEEEIREFEKEVK
cb4	SPVKRFIREVLEEAEEAYEGGDRHQFEELLWLANWAARDANDEEAEEEIREFEKEVK
cb6	SGAKRFVREVLEEAEEAYEKGDRRQFEELLWLAQWAARDANDEELEEEIREFEKEVK
Trimer1 (SB175[24])	SEALEELEKALRELKKSTDELERSTEELEKNPSEDALVENNRLIVENNKIIVEVLRIIAKVLK
Trimer2 (HALC3_104[37])	KRIDEIESKLKHLEEFTTHLIKLMETMLELLKLVSDGKSDSEEYKELLEKAEEYLKQATEAAKKI
Trimer3 (HALC3_110[37])	LEQILEELTELLERVDEIPLREALKRMLELLVRVTQELKEVKDKVESLEKHLEELDKRVEEIEKK
Trimer4 (HALC3_114[37])	VDEKEVKERFEEIESRLEELESKVREVEKKVEEVKKESDEKIDQLKTEFETKYNQINNEINTLKN
Trimer5 (HALC3_118[37])	MTRLEQLLAQGVDPFEVLREKIEKLKEIWKKYEEAKGEEKERYRDELLKLMMEVLELMVELLSRR
Trimer6 (HALC3_919)	SEELLEELRELLERLQELLELIEQGKITPEQLREAIALLIEVLQILYEALRELAEQLQRLREELG
nTrimer1	cb3-GSG-trimer1
nTrimer1_linker 1	cb3-GS-trimer1
nTrimer1_linker 2	cb3-GSGS-trimer1
nTrimer1_linker 3	cb3-GSGSGS-trimer1
nTrimer1_linker 4	cb3-GSGSGSGS-trimer1
nTrimer1_linker 5	cb3-GSGSGSGSGS-trimer1
nTrimer1_linker 6	cb3-GGGS-trimer1
nTrimer1_linker 7	cb3-GGGSGGGS-trimer1
nTrimer1_linker 8	cb3-GGGSGGGSGGGS-trimer1

Table S5 related to Figure 2. Cryo-EM data collection and refinement statistics.

**Data collection and processing**	**MERS-CoV S in complex with nTrimer1_linker 1 (Global refinement, 2 RBDs engaged)**	**MERS-CoV S in complex with nTrimer1_linker 1 (Global refinement, 3 RBDs engaged)**	**MERS-CoV S in complex with nTrimer1_linker 7 (Global refinement)**	**MERS-CoV S in complex with nTrimer1_linker 7 (Local refinement)**	**MERS-CoV S in complex with nTrimer1_linker 7 (Global refinement after focused classification, 3 RBDs engaged)**
Magnification	105,000	105,000	105,000	105,000	105,000
Voltage (kV)	300	300	300	300	300
Electron exposure (e^{-}/Å^2)	53.25	53.25	53.25	53.25	53.25
Defocus range (μm)	-0.9/ -1.6	-0.9/ -1.6	-0.6/ -1.25	-0.6/ -1.25	-0.6/ -1.25
Pixel size (Å)	0.829	0.829	0.829	0.829	0.829
Symmetry imposed	C1	C1	C1	C1	C1
Initial number of particles	615,255	615,255	986,712	986,712	986,712
Final number of particles	271.037	79.019	538.654	169.944	63.523
Map resolution (Å)	2.9	3.3	2.6	3.2	3.0
FSC threshold	0.143	0.143	0.143	0.143	0.143
Map sharpening B factor (Å^2)	73.79	19.11	76.83	-19.96	-21.03
**Validation**					
MolProbity score	N/A	N/A	N/A	1.25	N/A
Clash score	N/A	N/A	N/A	4.75	N/A
Poor rotamers (%)	N/A	N/A	N/A	0.00	N/A
**Ramachandran plot**					
Favored (%)	N/A	N/A	N/A	98.56	N/A
Allowed (%)	N/A	N/A	N/A	1.44	N/A
Disallowed (%)	N/A	N/A	N/A	0.0	N/A
**Entry codes**	EMD:46947	EMD:46952	EMD:46955	PDB: 9DKK<br>EMDB: 46960	EMD:46957
