# Supplementary figures and images for: Designed miniproteins potently inhibit and protect against MERS-CoV
Source: Cell Rep. Author manuscript; Available in PMC 2025 Jul 20. (PMC12276895; doi:10.1016/j.celrep.2025.115760)

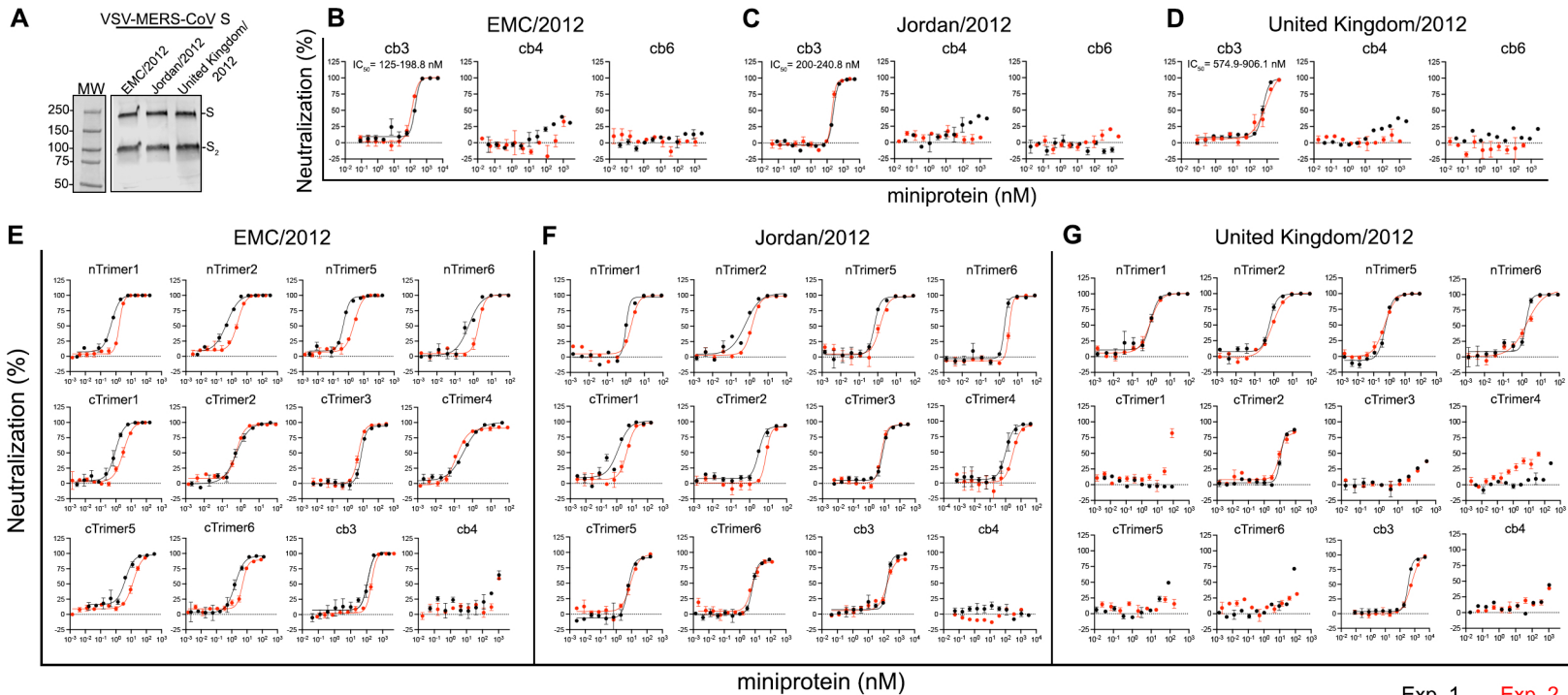

Supplement: FigureS1 — Fig S1. Inhibition of MERS-CoV S-mediated entry into VeroE6-TMPRSS2 cells by monomeric and trimeric designed miniproteins, related to Figure 1B. A, Western blot analysis of VSV pseudotyped particles harboring MERS-CoV EMC/2012, Jordan/2012 or United Kingdom/2012 S detected using the B6 stem-helix monoclonal antibody49 as a primary antibody. Full-length S and S2 subunit bands are indicated on the right-hand side of the blot. B-D, Concentration-dependent inhibition of MERS-CoV S pseudovirus entry into VeroE6-TMPRSS2 cells for MERS-CoV S EMC/2012 (B), Jordan/2012 (C) and United Kingdom/2012 (D) by monomeric miniproteins. E-F, MERS-CoV EMC/2012 (E), Jordan/2012 (F) and United Kingdom/2012 (G) S VSV pseudovirus-mediated entry in the presence of various dilutions of the indicated trimeric miniproteins. Monomeric miniprotein cb3 was used as a reference and cb4 as negative control of neutralization. Exp. 1 and Exp. 2 correspond to two biological experiments performed with two different preparations of pseudotyped viruses and miniproteins. Error bars represent the standard error of the mean (SEM) of the technical duplicates. Fits are shown only when neutralization is detected. [file NIHMS2090671-supplement-FigureS1.pdf]

**A**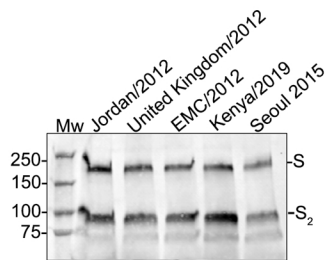**C**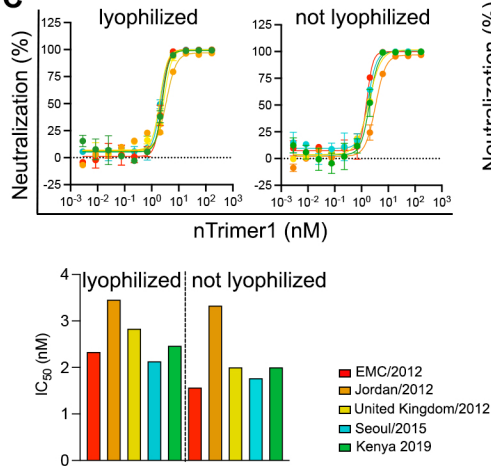**B**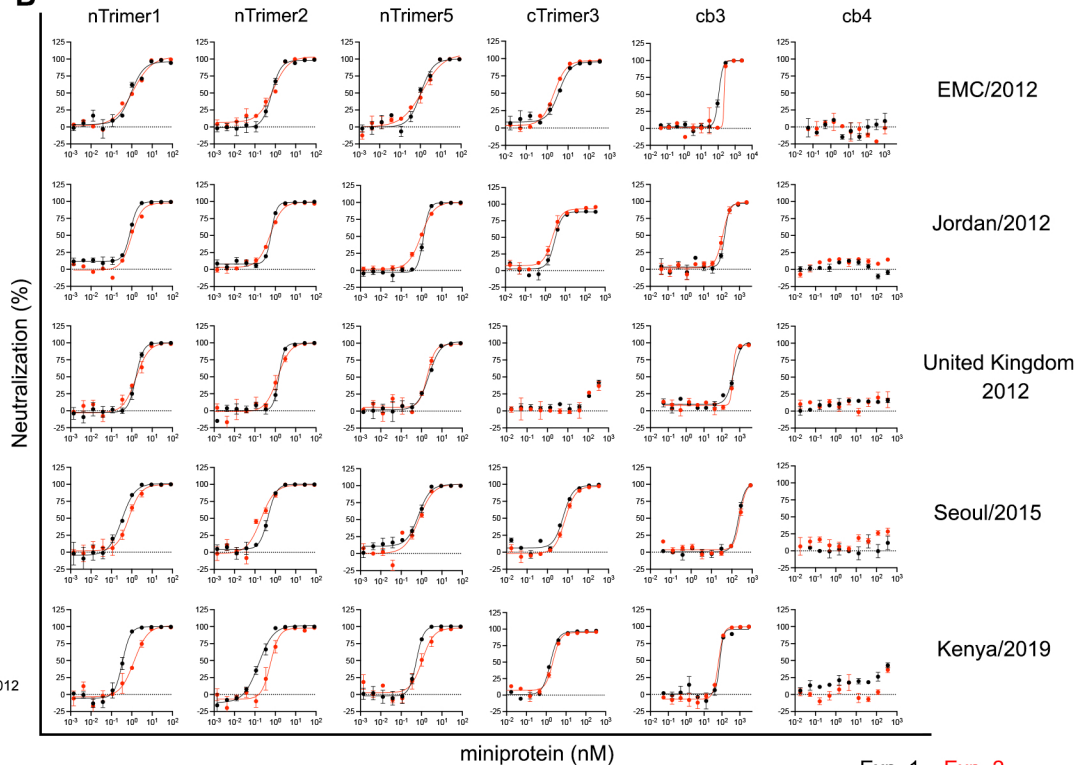

Supplement: FigureS2 — Fig S2. Inhibition of VSV-pseudotyped MERS-CoV S-mediated entry by nTrimer1, related to Figure 1B and Table 1. A. Western blot analysis of VSV pseudotyped particles harboring the indicated MERS-CoV S variants detected using the stem-helix monoclonal antibody B649 as a primary antibody. Mw, molecular weight ladder. Full-length S and S2 subunit bands are indicated on the right-hand side of the blot. B. MERS-CoV EMC2012, Jordan/2012, United Kingdom/2012, Kenya/2019 and Seoul/2015 S VSV pseudovirus entry in the presence of various dilutions of the indicated miniproteins. Exp. 1 and Exp. 2 correspond to two biological experiments performed with two different preparations of pseudotyped viruses and miniproteins. Error bars represent the standard error of the mean (SEM) of technical duplicates. Fits are shown only when neutralization is detected. C. MERS-CoV EMC/2012, Jordan/2012, United Kingdom/2012, Kenya/2019 and Seoul/2015 S pseudovirus entry in the presence of various dilutions of nTrimer1 lyophilized and reconstituted or not lyophilized. A single biological experiment with technical duplicates is shown. Error bars represent the standard error of the mean (SEM) of the technical duplicates. IC50 values, expressed in nanomolar, obtained from the experiment shown in the top panels. [file NIHMS2090671-supplement-FigureS2.pdf]

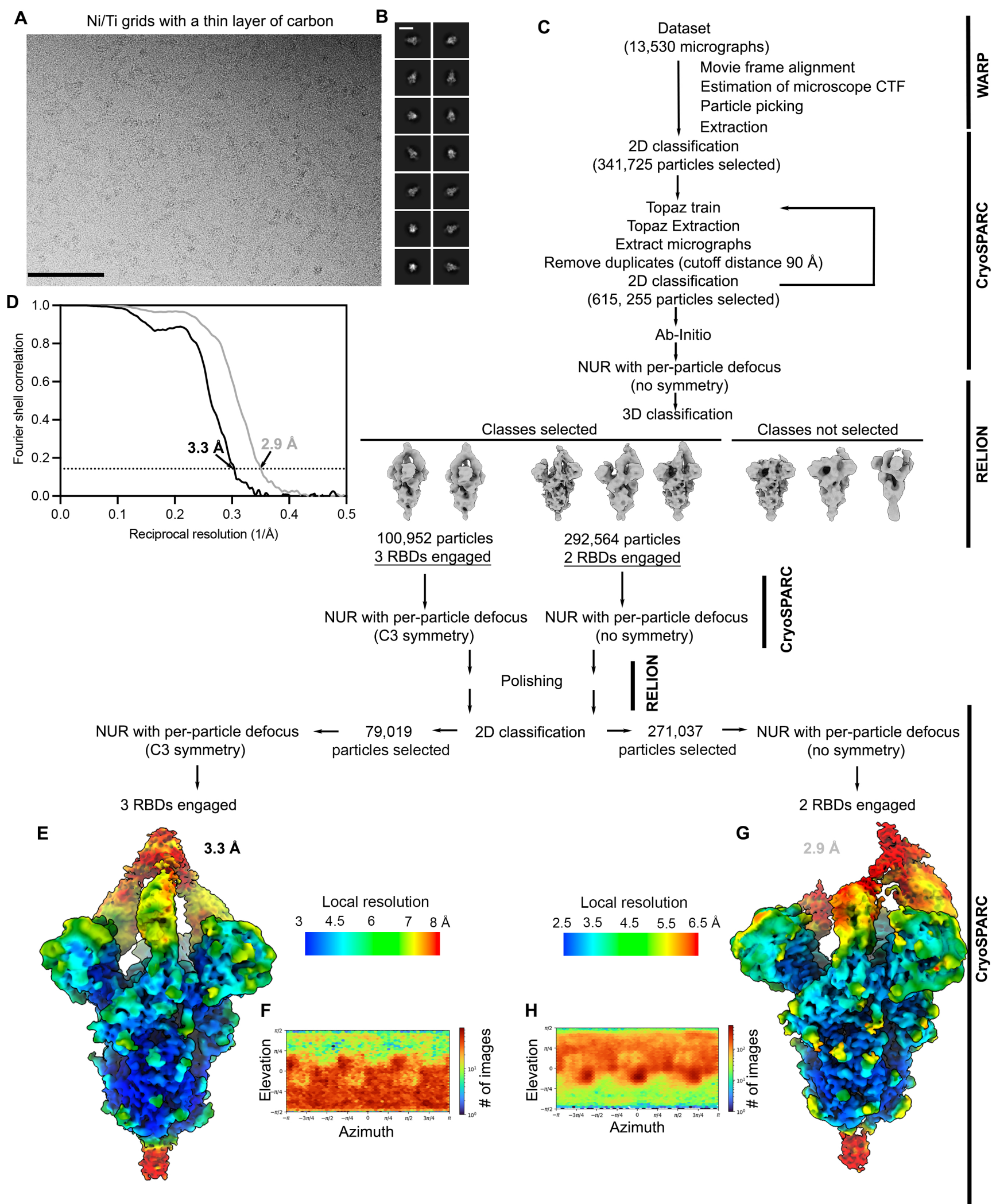

Supplement: FigureS3 — Fig S3. CryoEM data processing and validation of the structure of MERS-CoV S in prefusion conformation in complex with nTrimer1 (cb3-GSG-Trimer1), related to Figure 2E. A. Representative electron micrograph. B. 2D class averages. Scale bar of the micrograph and the 2D class averages, 100 nm and 100 Å, respectively. C. Cryo-EM data processing flowchart. CTF: contrast transfer function. NUR: non uniform refinement. D. Gold-standard Fourier shell correlation curves for the global maps with three and two RBDs engaged are shown in black and gray, respectively. The 0.143 cutoff is indicated by a horizontal dotted black line. E. Unsharpened map corresponding to prefusion MERS-CoV S in complex with three nTrimer1 colored by local resolution. F. Angular distribution plot with all the particles contributing to the map in E. G. Unsharpened map corresponding to the prefusion MERS-CoV S in complex with two nTrimer1 colored by local resolution. H. Angular distribution plot with all the particles contributing to the map in G. [file NIHMS2090671-supplement-FigureS3.pdf]

**A**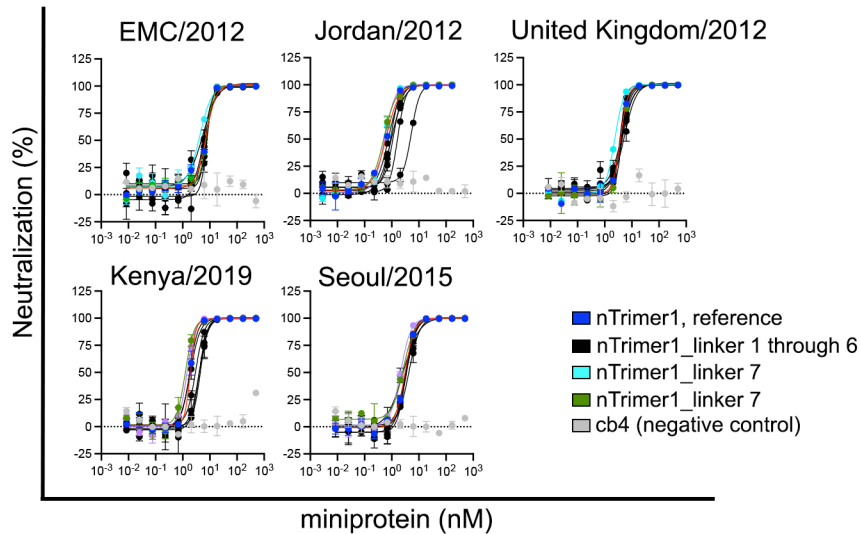**B**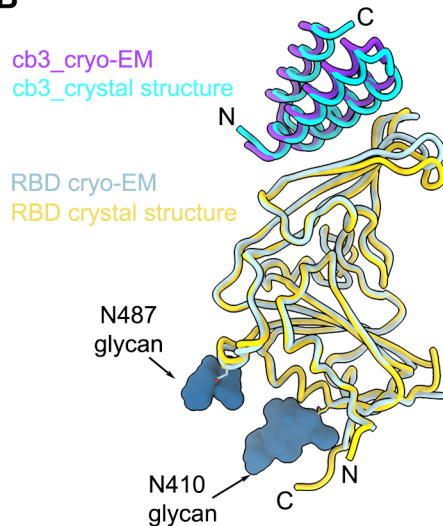

Supplement: FigureS4 — Fig S4. Optimization of the linker length between the miniprotein binding domain cb3 and trimer1 A-B, related to Figure 2. A. MERS-CoV EMC/2012, Jordan/2012, United Kingdom/2012, Kenya/2019 and Seoul/2015 S VSV pseudovirus entry into cells in the presence of various dilutions of nTrimer1 with different linkers lengths between cb3 and trimer1. Miniprotein cb4 was used as a negative control. A single biological experiment with two technical replicates is shown. Error bars represent the standard error of the mean (SEM) of the technical duplicates. B. Structural overlay between the cryo-EM structure of the MERS-CoV S RBD in complex with nTrimer1 linker 7 and the X-ray structure of the MERS-CoV S RBD in complex with cb3. [file NIHMS2090671-supplement-FigureS4.pdf]

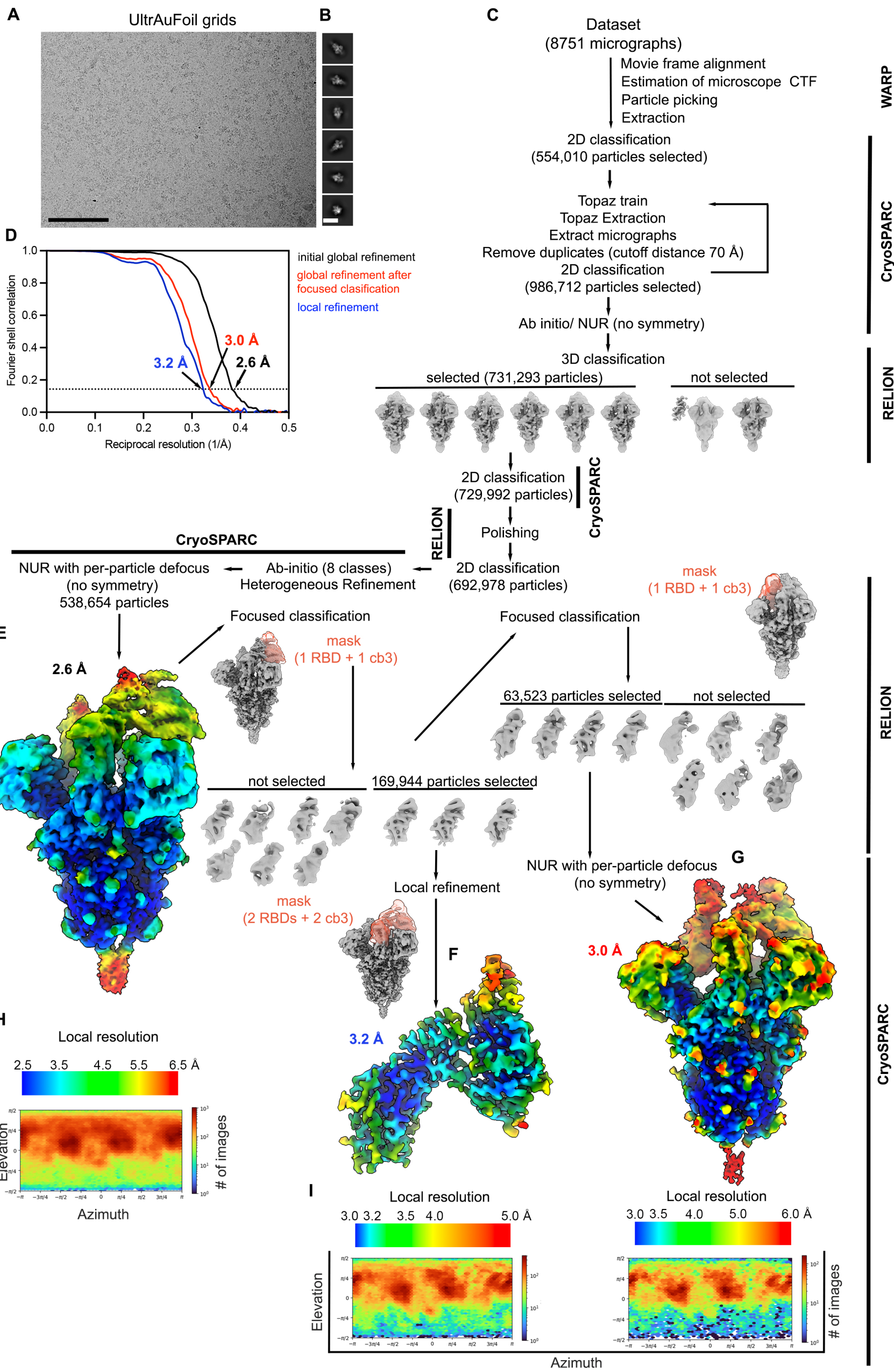

Supplement: FigureS5 — Fig S5. CryoEM data processing and validation of the structure of prefusion MERS-CoV S in complex with nTrimer1_linker 7 (cb3_GGGSGGGS_trimer1), related to Figure 2. A. Representative electron micrograph. B. 2D class averages. Scale bar of the micrograph and the 2D class averages, 100 nm and 100 Å, respectively. C. Cryo-EM data processing flowchart. CTF: contrast transfer function. NUR: non uniform refinement. D. Gold-standard Fourier shell correlation curves for the global maps (black and red) and locally refined map (blue). The 0.143 cutoff is indicated by a horizontal dotted black line. E. Unsharpened map corresponding to the 3D reconstruction of MERS-CoV S (in prefusion conformation) in complex with nTrimer1_linker 7 colored by local resolution. F. Locally refined sharpened map corresponding to two neighboring MERS-CoV S RBDs each engaging one cb3 from the nTrimer1_linker 7 miniprotein colored by local resolution. G. Global unsharpened map for the MERS-CoV-S in complex with nTrimer1_linker 7 miniprotein obtained after focused classification and colored by local resolution. H, I. Angular distribution plots corresponding to the maps shown directly above. [file NIHMS2090671-supplement-FigureS5.pdf]
