# Supplementary material for: Designed miniproteins potently inhibit and protect against MERS-CoV
Source: Cell Rep. Author manuscript; Available in PMC 2025 Jul 20. (PMC12276895; doi:10.1016/j.celrep.2025.115760)
Supplement: StarMethodsTable — Table S1 related to Figure 1F–G. X-ray crystallography data collection and refinement statistics of the cb3-MERS-CoV RBD complex. Table S2. List of IC50 values obtained from the neutralization curves shown in Fig S1. Monomeric miniprotein cb3 was used as a reference to highlight the improved neutralization exhibited by the trimerization of cb3. Monomeric miniprotein cb4 was used as a negative control. The two IC50 values for each pseudotyped virus correspond to two distinct biological experiments performed with two batches of pseudovirus and one batch or miniprotein. The “n” and “c” indices refer to the position N- or C-terminus of the miniprotein cb3 relative to the indicated trimerization domain. Limit of detection (LOD) of neutralization is between 5×10^2^-103 nM (see Fig S1). Table S3 related to Figures 1E and 2C. Summary of binding kinetics for monomeric and trimeric miniproteins to the prefusion MERS-CoV S trimer. KD or apparent KD (KDapp denoted with a star, due to multivalent binding and avidity) values were determined through global langmuir 1:1 model fitting. Table S4. Sequences of miniproteins, trimerization domains, and fusion expression constructs. Each trimerization domain was tested as an N- and C-terminal fusion with respect to the miniprotein cb3. All constructs were expressed with MSG - design - GS - SNAC tag - 6x his, as described in methods. The name in brackets indicates a previously published name for that homotrimer domain. Table S5 related to Figure 2. Cryo-EM data collection and refinement statistics. [file NIHMS2090671-supplement-StarMethodsTable.docx]

## **STAR Methods Table**

| REAGENT or RESOURCE | SOURCE | IDENTIFIER |
| --- | --- | --- |
| **Antibodies** | | |
| B6 monoclonal antibody (anti-stem helix) | Sauer *et al.*[*^49^*](https://paperpile.com/c/OWGOED/3rcW) | N/A |
| AF680 conjugated goat anti-human | Jackson ImmunoResearch | 109-625-098 |
| JC57-11 monoclonal antibody (anti-RBD) | Tse *et al.*[*^21^*](https://paperpile.com/c/OWGOED/Myts) | N/A |
| Anti-myc FITC | Immunology Consultants Laboratory | cat# CMYC-45F |
| Anti-cleaved caspase 3 (Asp175) | Cell Signaling Technology | cat# 9661 |
| Anti-MERS-CoV nucleocapsid polyclonal sera | Cockrell *et al.*[*^42^*](https://paperpile.com/c/OWGOED/4pQdo) | N/A |
| **Bacterial and virus strains** | | |
| BL21 (DE3) | NEB | cat# C2527I |
| *S. cerivasiae* (EBY100) | Yeast Resource Centre, University of Washington | EBY100 |
| VSV (G*∆G-luciferase) | Kaname et al.[^66^](https://paperpile.com/c/OWGOED/7UKx) | N/A |
| MERS-CoV EMC 2012 nanoluciferase reporter virus | This work | N/A |
| Mouse adapted MERS-CoV EMC 2012 (m35c4) | This work | N/A |
| **Chemicals, peptides, and recombinant proteins** | | |
| MERS-CoV S RBD | Addetia *et al.* 2024[^9^](https://paperpile.com/c/OWGOED/hQoTl) | N/A |
| MERS-CoV S trimer | Park *et al.*[*^13^*](https://paperpile.com/c/OWGOED/wnPSj) | N/A |
| MERS-CoV S trimer biotinylated | Acro Bio | cat# SPN-M82E3 |
| Human DPP4 | Addetia *et al.* 2024[^9^](https://paperpile.com/c/OWGOED/hQoTl) | N/A |
| HA miniprotein | Cao *et al.*[*^25^*](https://paperpile.com/c/OWGOED/GIVk) | N/A |
| Streptavidin-PE | Thermo Fisher | cat# S866 |
| BirA biotin-protein ligase standard reaction kit | Avidity | cat# BirA500 |
| cb3 | This work | N/A |
| cb4 | This work | N/A |
| cb6 | This work | N/A |
| nTrimer1 | This work | N/A |
| nTrimer2 | This work | N/A |
| nTrimer5 | This work | N/A |
| nTrimer6 | This work | N/A |
| cTrimer1 | This work | N/A |
| cTrimer2 | This work | N/A |
| cTrimer3 | This work | N/A |
| cTrimer4 | This work | N/A |
| cTrimer5 | This work | N/A |
| cTrimer6 | This work | N/A |
| nTrimer1 linker 1 | This work | N/A |
| nTrimer1 linker 2 | This work | N/A |
| nTrimer1 linker 3 | This work | N/A |
| nTrimer1 linker 4 | This work | N/A |
| nTrimer1 linker 5 | This work | N/A |
| nTrimer1 linker 6 | This work | N/A |
| nTrimer1 linker 7 | This work | N/A |
| nTrimer1 linker 8 | This work | N/A |
| **Critical commercial assays** | | |
| Biotin capture kit (SPR) | Cytiva | cat# 28920234 |
| Nano-Glo® Luciferase Assay System | Promega | cat# N1130 |
| **Deposited data** | | |
| MERS-CoV RBD | PDB | 4L3N |
| MERS-CoV RBD | PDB | 4KR0 |
| **Experimental models: Cell lines** | | |
| HEK293T | ATCC | cat# CRL-3216 |
| VeroE6-TMPRSS2 | JCRB-Cell bank | cat# JCRB1918 |
| Expi293F | Thermo Fisher | cat# A14527 |
| VeroE6-TMPRSS2-GFP_11_ | Addetia et al., 2023[^67^](https://paperpile.com/c/OWGOED/RHZx) | N/A |
| BHK-21-GFP_1-10_ | Addetia et al., 2023[^67^](https://paperpile.com/c/OWGOED/RHZx) | N/A |
| Huh7.5 | Blight *et al.*[*^85^*](https://paperpile.com/c/OWGOED/ZzWb) | N/A |
| **Experimental models: Organisms/strains** | | |
| 288/330 C57BL/6J mice | Cockrell *et al.*[*^42^*](https://paperpile.com/c/OWGOED/4pQdo) | N/A |
| **Recombinant DNA** | | |
| pcDNA3.1 (+) MERS-CoV S Jordan/2012 full-length | GenScript | N/A |
| pcDNA3.1(+) MERS-CoV EMC/2-12 S full-length | Addetia *et al.*, 2024[^9^](https://paperpile.com/c/OWGOED/hQoTl) | N/A |
| pcDNA3.1(+) MERS-CoV United Kingdom/2012 S full-length | Addetia *et al.*, 2024[^9^](https://paperpile.com/c/OWGOED/hQoTl) | N/A |
| pcDNA3.1(+) MERS-CoV Seoul/2015 S full-length | Addetia *et al.*, 2024[^9^](https://paperpile.com/c/OWGOED/hQoTl) | N/A |
| pcDNA3.1(+) MERS-CoV Kenya/2019 S full-length | Addetia *et al.*, 2024[^9^](https://paperpile.com/c/OWGOED/hQoTl) | N/A |
| B6 plasmid | Sauer *et al.*[*^9^*](https://paperpile.com/c/OWGOED/hQoTl) | N/A |
| LM0627 cb3 | This work | N/A |
| LM0627 cb4 | This work | N/A |
| LM0627 cb6 | This work | N/A |
| pET29b+ nTrimer1 | This work | N/A |
| pET29b+ nTrimer2 | This work | N/A |
| pET29b+ nTrimer5 | This work | N/A |
| pET29b+ nTrimer6 | This work | N/A |
| pET29b+ cTrimer1 | This work | N/A |
| pET29b+ cTrimer2 | This work | N/A |
| pET29b+ cTrimer3 | This work | N/A |
| pET29b+ cTrimer4 | This work | N/A |
| pET29b+ cTrimer5 | This work | N/A |
| pET29b+ cTrimer6 | This work | N/A |
| LM0627 nTrimer1 linker 1 | This work | N/A |
| LM0627 nTrimer1 linker 2 | This work | N/A |
| LM0627 nTrimer1 linker 3 | This work | N/A |
| LM0627 nTrimer1 linker 4 | This work | N/A |
| LM0627 nTrimer1 linker 5 | This work | N/A |
| LM0627 nTrimer1 linker 6 | This work | N/A |
| LM0627 nTrimer1 linker 7 | This work | N/A |
| LM0627 nTrimer1 linker 8 | This work | N/A |
| pETcon3 yeast display design library | This work | N/A |
| **Software and algorithms** | | |
| Rosetta | RosettaCommons | <https://rosettacommons.org/software/> |
| Biacore Evaluation software | Cytiva | <https://www.cytivalifesciences.com/en/us/shop/protein-analysis/spr-label-free-analysis/spr-software-and-extensions/biacore-insight-evaluation-software-p-23528> |
| Prism 10 | GraphPad | <https://www.graphpad.com/> |
| Gen5 image prime v3.11 | Agilent | <https://www.agilent.com/en/product/microplate-instrumentation/microplate-instrumentation-control-analysis-software/imager-reader-control-analysis-software> |
| PyMol 3 | Schrödinger LLC | <https://pymol.org/> |
| UCSF Chimera X | Meng *et al.*[*^82^*](https://paperpile.com/c/OWGOED/k9VQ) | <https://www.rbvi.ucsf.edu/chimerax/> |
| Molprobity | Williams *et al.*[*^63^*](https://paperpile.com/c/OWGOED/AcTl3) | http://molprobity.biochem.duke.edu/ |
| Privateer | Agirre *et al.*[*^64^*](https://paperpile.com/c/OWGOED/d5t3) | https://privateer.york.ac.uk/ |
| Phenix-Refine |  | https://phenix-online.org/documentation/index.html |
| Phenix-Phaser | McCoy *et al.*[*^59^*](https://paperpile.com/c/OWGOED/22dFg) | https://phenix-online.org/documentation/index.html |
| Coot | Emsley *et al.*[*^61^*](https://paperpile.com/c/OWGOED/nuOJJ) | https://www2.mrc-lmb.cam.ac.uk/personal/pemsley/coot/ |
| CryoSPARC | Punjani *et al.*[*^71^*](https://paperpile.com/c/OWGOED/tyzVj) | <https://cryosparc.com/> |
| AlphaFold2 MCMC hallucination | Wicky *et al.*[*^37^*](https://paperpile.com/c/OWGOED/u0AjL) | https://github.com/bwicky/oligomer_hallucination |
| ProteinMPNN | Dauparas *et al.*[*^52^*](https://paperpile.com/c/OWGOED/3ZHH) | https://github.com/dauparas/ProteinMPNN |
| DNAworks 2.0 | Hoover *et al.*[*^55^*](https://paperpile.com/c/OWGOED/7kNP) | https://github.com/davidhoover/DNAWorks |
